# Influence of living in the same home on biomonitored levels of consumer product chemicals

**DOI:** 10.1038/s41370-021-00368-8

**Published:** 2021-07-13

**Authors:** Robin E. Dodson, R. Woodrow Setzer, John D. Spengler, Julia G. Brody, Ruthann A. Rudel, Jose Guillermo Cedeño Laurent

**Affiliations:** 1 Silent Spring Institute, Newton, MA USA; 2 Retired, Chapel Hill, NC USA; 3 Harvard T.H. Chan School of Public Health, Boston, MA USA

**Keywords:** Biomonitoring, Chemicals in Products, Endocrine Disruptors, Empirical/Statistical Models

## Abstract

**Background:**

Individuals living in the same home may share exposures from direct contact with sources or indirectly through contamination of the home environment.

**Objective:**

We investigated the influence of sharing a home on urine levels of ten phenolic chemicals present in some consumer products.

**Methods:**

We used data from Silent Spring Institute’s Detox Me Action Kit (DMAK), a crowdsourced biomonitoring program in the US. Of the 726 DMAK participants, 185 lived in the same home with one or more other DMAK participants (*n* = 137 pairs, up to six participants in a home). The concentration distributions included values below the detection limit so we used statistical methods that account for left-censored data, including non-parametric correlation estimation and hierarchical Bayesian regression models.

**Results:**

Concentrations were significantly positively correlated between pair-members sharing a home for nine of the ten chemicals. Concentrations of 2,5-dichlorophenol were the most strongly correlated between pair-members (tau = 0.46), followed by benzophenone-3 (tau = 0.31) and bisphenol A (tau = 0.21). The relative contribution of personal product use reported product use of other household members (up to 5 others), and the residual contribution from a shared household, including exposures not asked about, varied by chemical. Paraben concentrations were largely influenced by personal behaviors whereas dichlorophenol and bisphenol concentrations were largely influenced by shared home exposures not related to reported behaviors.

**Significance:**

Measuring the influence of personal and household practices on biomonitoring exposures helps pinpoint major sources of exposure and highlights chemical-specific intervention strategies to reduce them.

## Introduction

Many consumer products are used in homes resulting in chemical exposures by contact, inhalation directly, or after partitioning to suspended aerosols and dust. Individuals living in the same home may share these exposures and/or may be exposed differently through personal use of products. Disentangling the sources of exposure is important for developing effective intervention strategies to reduce consumer product chemical exposures.

Several phenolic compounds that are known to mimic estrogen or have other hormone activity are commonly used in consumer products. Parabens are preservatives in personal care products and pharmaceuticals, and may also be found in food, paper, plastics, paints, and building materials [1–4]. Benzophenone-3 is a UV filter used in personal care products, including as an active ingredient in sunscreen, and in plastics and protective coatings, including paint [1, 5]. Bisphenol A (BPA) is found in polycarbonate plastics, thermal papers, and epoxy resins, some of which are used in food can linings [4, 6–8]. Bisphenol F (BPF), a chemical analog to BPA with similar biological activity, is also used in epoxy resins and there is limited information on the extent of its uses. Bisphenol S (BPS), another analog to BPA, is used in thermal paper, including receipts, and food contact materials [7, 9, 10]. The antimicrobial triclosan was historically used in hand soaps until the US Food and Drug Administration ban in 2016 [11]. Triclosan may still be found in other personal care products like toothpaste and deodorant and in building materials like countertops, flooring, and bathroom fixtures [1, 12]. 2,4-Dichlorophenol is a minor degradation product of triclosan and is also a metabolite of 2,4-D, an herbicide, and of the wood preservative pentachlorophenol [13]. Finally, 2,5-dichlorophenol is a metabolite of 1,4-dichlorobenzene, a disinfectant and pesticide used in mothballs and deodorizers, and may have other uses [14]. Both 2,4-dichlorophenol and 2,5-dichlorophenol have been identified as possible drinking water contaminants [15, 16].

These phenolic chemicals have been measured in air and dust in homes [17–19]. Because these chemicals are semivolatile organic compounds, they readily partition between the gaseous and particulate phase throughout the home so can be measured in air, dust, and on surfaces. Parabens and BPA have been measured in indoor air in homes [17]. We measured parabens, BPA, and triclosan in the air in newly renovated subsidized housing units in Boston, MA, and found that their levels increased with occupancy [18]. Parabens and BPA have also been detected in household dust [19]. The presence of these chemicals in indoor air and household dust indicates that the home environment can be a source of exposure.

After exposure, these chemicals are rapidly metabolized and can be readily measured in urine. Data from the National Health and Nutrition Examination Survey (NHANES) indicate that nearly all Americans have these chemicals in their urine [20]. Urine biomonitoring aggregates exposure from various sources, for example, diet, product use, and ambient environment, and consumer product use has been shown to be a strong predictor of biological levels for many of these chemicals [21–28].

Individuals may be exposed to these chemicals because of their own product use, because of product use of others in their shared home, and because of the presence of these chemicals in some building materials and furnishings in the shared home (see Table 1). The influence of co-exposures in a shared home environment on urine concentrations of these chemicals has been investigated among adults in two fertility studies and in studies involving mother–child pairs. Chung et al. investigated patterns of co-exposure for a range of endocrine disrupting chemicals, including these consumer product chemicals, among partners living in Michigan and Texas enrolled in the longitudinal investigation of fertility and the environment (LIFE) study [29]. The percent of variance explained by the shared home was less than 10% for phenols, including BPA, and just over 15% for antimicrobials, including triclosan and parabens. Among adult couples seeking fertility assistance in Boston, MA, correlations between partners (intra-class correlation coefficients (ICCs)) were between 0.4 and 0.6 for 2,5-dichlorophenol, triclosan, benzophenone-3, and 2,4-dichlorophenol, and ICCs/correlations were lower for bisphenols and parabens (range 0.15–0.3) [30]. Both of these studies relied on a single spot urine sample from each partner collected at the same time during the day, and so may not reflect exposure from the home because of the short biological half-lives of these chemicals, and neither study investigated behavior patterns associated with measured urine concentrations among couples. Studies of mother–child pairs have found a moderate positive correlation in urine phenol concentrations among the pair members, and some have found associations with mothers’ behavior [31].

**Table 1:** Expected major exposure sources for 10 phenolic chemicals.

Chemical group	Chemicals	Personal behavior-related sources			Shared home-based sources		
		Personal Care Products	Diet	Pharmaceuticals	Cleaning Products	Building Materials	Water
Parabens	methyl paraben (MePB)	✓	✓	✓			
	ethyl paraben (EtPB)	✓	✓	✓			
	propyl paraben (PrPB)	✓	✓	✓			
UV filter	benzophenone-3 (BP3)	✓	✓			✓	
Antimicrobial	triclosan (TCS)	✓			✓	✓	
Dichlorophenols	2,4-dichlorophenol (DCP24)				✓		✓
	2,5-dichlorophenol (DCP25)				✓		✓
Bisphenols	bisphenol A (BPA)		✓			✓	
	bisphenol S (BPS)		✓				
	bisphenol F (BPF)		✓				

We sought to investigate the influence of behaviors and home-based exposures on biomonitoring levels of 10 phenolic consumer product chemicals. We used data from Silent Spring Institute’s Detox Me Action Kit (DMAK), a crowdsourced national biomonitoring program. We included participants who shared a home with at least one other DMAK participant, which included adults of similar ages and different or same genders that may represent couples, and also adults and children and adults of different ages. We estimated the rank correlation of measured urine concentrations and the similarity of self-reported product use between pair-members. We developed a robust Bayesian hierarchical model to quantify the contribution of the shared home to measured concentrations and investigate the influence of personal behaviors and behaviors of others (up to 5 others) in the home on measured urine concentrations. Identifying the influence of behaviors and home-based exposures on biomonitoring levels can help develop effective strategies for limiting exposures to hormone-disrupting consumer product chemicals by highlighting the most significant sources of exposure for each chemical.

## Methods

### Identifying participants in shared homes

We used data from 726 participants in DMAK. DMAK is a crowdsourced—some participants and others financially support the project—biomonitoring program that recruited between December 2016 and October 2018 [32]. DMAK is designed to support exposure research and to improve environmental health literacy by allowing members of the public to learn about their chemical body burden. Participants collected their own urine samples and then shipped the frozen samples overnight to Silent Spring Institute where they were batched and sent to a contract lab for chemical analysis. Once results were available, participants received personalized study reports via Silent Spring Institute’s Digital Exposure Report-Back Interface (DERBI) [33]. Additional details about the study methods and results can be found in Dodson et al. [34].

We identified the subset of participants who shared home if they had the same mailing address where they received the urine collection kit as another participant in the study. Seventeen households joined the study specifically to investigate correlated exposures between two individuals in their households. We included only those participants who returned kits within 4 weeks of each other. We conducted analyses to evaluate the sensitivity of our results to the selection of a 4-week time period versus a 1-week time period.

### Sample and data collection

Urine collection kits were mailed to participants starting in February 2017 and the last kit was returned in October 2018. Kits included an insulated shipping box containing two 4 oz amber glass jars (Environmental Sampling Supply, #0125-0055-QC), instructions for sample collection, an overnight shipping label, ice packs, and shipping materials (materials available from authors upon request). Participants were instructed to collect two urine samples—one in the evening and the first void the following morning—and to freeze the samples and ice packs in their home freezer for at least 24 h before mailing them back via overnight mail. Samples were inspected at Silent Spring Institute and frozen at −20 °C until being sent to the analytical laboratory overnight on ice.

Participants were asked to complete an online questionnaire after collecting their morning urine sample. Questions were asked about demographics and product use within the last 24 h (dichotomous: yes/no). For example, participants were asked, “In the last 24 h, did you use this product?” Questions focused on personal behaviors, except for three questions related to use of a vacuum cleaner with HEPA filter in the home, use of damp cloth in the home, and application of weed killer to lawn or garden. Questions on product use were developed based a priori hypotheses about potential sources of phenolic consumer product chemicals (Table S1). Questionnaire is available upon request from the authors.

### Urine sample analysis

Samples were analyzed by two different laboratories to accommodate their availability; 101 samples were analyzed at NSF International (Ann Arbor, MI USA) and 84 samples were analyzed at SGS AXYS Analytical (Sidney, BC Canada). Samples from participants in the same household were always analyzed by the same laboratory. Upon receipt at the laboratories, urine samples were thawed and the two urine samples from each participant were composited in equal volumes to yield a single composite for each participant.

Chemical analysis details are provided elsewhere [34]. Briefly, target analytes were analyzed in urine samples using solid-phase extraction (SPE) coupled to high-performance liquid chromatography-isotope dilution tandem mass spectrometry (HPLC-MS/MS). Analytical methods are similar to those used at CDC’s Environmental Health Laboratory [35].

### Data analysis

We prioritized three parabens (methylparaben, ethylparaben, propylparaben), UV filter benzophenone-3, three bisphenols (BPA, BPS, BPF), antimicrobial triclosan, and two dichlorophenols (2,4-dichlorophenol, 2,5-dichlorophenol) for data analysis because they had higher detection frequencies (>35% above method reporting limit (MRL)) than other target analytes. We only report BPF results from NSF since results from SGS SXYS were reported as estimated with uncertain accuracy. Because several analytes have values that fall below the analytical detection limit—less than 100% detection frequency—we relied on statistical approaches that account for left censoring. We used Kaplan–Meier estimation methods to estimate the mean, standard deviation, and median concentrations for each chemical accounting for non-detects using the NADA R package [36].

To provide comparable results to previous studies and to evaluate correlation among pairs of participants living in the same home, we estimated correlations of measured concentrations between pair members using Kendall’s tau beta rank correlation, which accounts for left-censored data [37]. Correlation estimates and *p*-values were obtained from 10,000 bootstrap replications, assuming a lognormal distribution and adjusting for ties. Correlations were estimated among all pairs as well as separately for adult–child pairs (except BPF due to a low number of simultaneous detects) and adult–adult pairs. We did not analyze additional pair types (e.g., same-gender adult-adult pairs) due to a small number of pairs.

We used our a priori hypotheses about exposure sources when known, for each chemical to develop our analysis plan. The analysis plan identifies products and behaviors that may contribute to exposure to each chemical. For example, benzophenone-3 may be present in sunscreen so we included sunscreen use in the previous 24 h as a predictor variable for that chemical. See Supplementary Information for the full list of expected exposure-related behaviors for each chemical (Table S1).

To evaluate whether pair members reported similar behaviors, and again to better understand whether there was concordance among participants within the same home, we estimated similarity between pairs for self-reported behaviors using the Jaccard coefficient. The Jaccard coefficient is the ratio of the intersection of the union between two respondents, with larger values indicating greater similarity. We used the centered Jaccard coefficient, the Jaccard coefficient minus its expectation under independence. We estimated 95% confidence intervals for the centered Jaccard estimates using 1,000 bootstraps and not adjusting for multiple comparisons. Of note, we did not collect information to determine whether participants used the same specific product brand name. While parents completed the product use survey for their children, they were asked to answer questions based on their child’s behaviors and not theirs.

To quantify the relative importance of personal product use, use of products by others in the home, and a shared home on each participant’s urine concentration, we used a Bayesian hierarchical model. We relied on data from participants sharing a home (including when more than two participants shared a home) as well as data from the full cohort. We assumed that an individual’s urine concentration results from the sum of contributions from: individual product use behaviors, product use behaviors reported by other household members, and household background, which includes the use of products not asked about. Urine concentrations were non-negative, and their distribution tended to be right-skewed. Typically, concentration data such as these are modeled as lognormally distributed with estimated log median and shape parameters. We followed that convention except that, here, the *mean* of the data distribution was modeled as the sum of elements reflecting the partitioning of the contributions listed above, in contrast to the usual practice of modeling the logarithm of the median of the data distribution as the sum of elements. We used the mean in place of the more usual parameterization of the lognormal distribution because we wanted to partition exposure into components due to different sources: common sources within the household, personal use of products, and use of products by other household members. Such contributions would add together to form the mean of the overall distribution. Further, both to regularize the estimates and to estimate the variance among households, the household contribution was modeled as a lognormal random variable with unknown parameters. We computed a single intercept term for participants in shared homes and then assigned separate shape parameters to the data distributions for participants in shared homes and participants in the rest of the DMAK cohort (single participant per home). We assumed the contribution of personal product use to be the same over both sets of participants, allowing both parts of the data set to be used to estimate contributions of personal product use behaviors. Observations below their MRL were treated as left-censored when calculating their contributions to the overall likelihood. We used the R package “rstan” to estimate model parameters using Bayesian methods, yielding point estimates (posterior means and medians) and credible intervals [38, 39]. See Supplementary Information for further details, including model equations, and Stan code.

## Results and discussion

### Participant characteristics

Of the 726 DMAK participants, 185 shared a home with one or more other DMAK participants, with a total of 82 homes and 137 unique pairs (e.g., a family of three might include parent 1-child, parent 1-parent 2, child-parent 2 in the same home). Most (82%) of the shared homes had two participants, 11% of homes had three participants, one home had four participants, two homes had five participants, and one home had six participants. Of the 137 pairs with urine data, we had demographic and product use data from the survey from 87 pairs. In addition to homes with similar-aged adults (≥18 years old), some homes were multigenerational (i.e., older adults, adults, and/or children). There were 26 adult–child pairs and seven child–child pairs. Of the 54 adult–adult pairs, 43 were male–female pairs, 10 were female–female pairs, and none were male–male pairs (one participant did not provide a gender). Seven pairs included an adult over 65 years old. The maximum age difference between pairs was 51 years (54-year-old adult with 3-year-old child) and 40 pair members were within 5 years of age with the other pair member.

### Measured concentrations

Observations for the overall study population are fully described in Dodson et al. [34]. Concentrations were similar among the subset of participants sharing a home with at least one other participant (*n* = 185) and the entire DMAK cohort. In this shared home subset, methylparaben and benzophenone-3 were detected in all of the urine samples and were also measured at the highest mean concentrations (Table 2). Ethyl and propylparaben were found at slightly lower levels, although still found in the majority of participants. While all concentrations distributions were skewed right, triclosan concentrations, in particular, were highly skewed with an estimated mean concentration two orders of magnitude higher than the estimated median and the 99th percentile one order of magnitude higher than the 95th percentile. BPA, BPF, and BPS were detected in the majority of participants, with BPF at the highest mean concentration of the three. Finally, 2,4-dichlorophenol and 2,5-dichlorophenol concentrations were generally in the 0.1–10 ng/ml range, with 2,5-dichlorophenol concentrations approximately one order of magnitude higher than 2,4-dichlorophenol concentrations.

**Table 2:** Urine concentrations (ng/ml) of phenolic compounds measured in participants sharing a home with another DMAK participant.

Chemical	*N*	% > MRL^a^	MRL^b^	Mean^c^	SD^c^	Median^c^	95th percentile	99th percentile	Range^d^
methylparaben	185	100	0.77	59.8	140	13.8	247	661	1.09–1080
ethylparaben	185	83	1	8.65	19.3	1.52	37.8	88	0.222–169
propylparaben	185	92	0.2	13.4	41	1.39	57.3	214	0.089–338
benzophenone-3	185	100	0.28	104	460	20.7	240	1870	0.639–5410
triclosan	185	34	1.8	43.4	301	0.63	134	550	0.691–3680
2,4-dichlorophenol	185	59	0.1	0.684	1.26	0.325	3.24	6.1	0.11–9.3
2,5-dichlorophenol	185	59	0.2	3.41	10.5	0.96	13.4	52.2	0.22–96.2
bisphenol A	185	76	0.28	1.35	2.5	0.663	4.3	12.9	0.207–24.2
bisphenol F^5^	101	89	0.24	2.4	9.59	0.72	4.44	49.5	0.25–83.4
bisphenol S	185	88	0.28	0.909	1.69	0.53	2.37	5.98	0.107–17

^a^Percent of samples above sample-specific method reporting limit (MRL).

^b^Median MRL.

^c^Kaplan–Maier non-parametric descriptive statistics method for left-censored data.

^d^Range of detected concentrations.

^e^Bisphenol *F* values from SGS AXYS dropped due to quality assurance/quality control issues.

### Concentration correlation among pairs

**Fig. 1:**
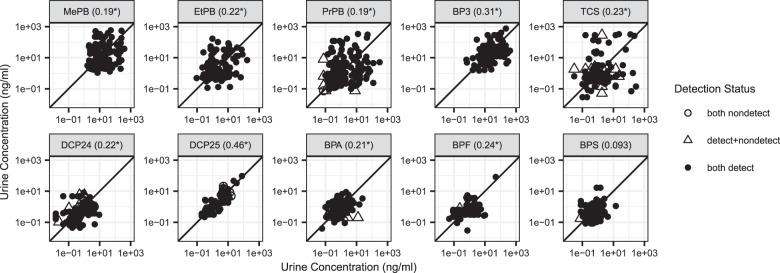
Scatterplots of measured urinary concentrations for each pair (1 pair member on *x*-axis and 1 pair member on *y*-axis). Note the logarithmic scale. 1:1 line shown. Point shapes indicate whether both values were above the MRL, one value was above and one value was below the MRL, or both were below the MRL. Values below the MRL are plotted at the MRL. Kendall’s tau beta correlation estimates in parentheses. Asterisks indicate a significant correlation (*p* < 0.05).

Concentrations between pair-members were significantly positively correlated for nine of the ten chemicals (Fig. 1). Concentrations of 2,5-dichlorophenol were the most strongly correlated between pair-members (tau = 0.46), followed by benzophenone-3 (tau = 0.31) and BPA (tau = 0.21). 2,5-Dichlorophenol is a metabolite of the deodorizer and pesticide 1,4-dichlorobenzene, and is likely used within the house rather than personally so use of the product at home will likely affect all residents. In a previously reported study of male–female adult partners, 2,5-dichlorophenol concentrations were also moderately correlated (spearman rho ~0.6) [30]. Benzophenone-3 is used in personal care products such as sunscreen to provide UV protection to the consumer [1]. It also has other uses, including in plastics and paints and on textiles, to protect the products from UV rays [4]. These other uses may be contributing to the correlation between pair-members. Benzophenone-3 was also moderately correlated between adult male–female partners in a fertility study (rho ~0.5) [30]. BPA correlation between pair-members suggests possible shared dietary sources or may be a result of its reported use in building materials such as paints, sealants, and adhesives [4]. Correlation estimates among pair-members indicate that participants sharing the same home may share exposures and provide context for the more comprehensive Bayesian hierarchical model results that account for multiple participants within a home.

We examined correlations among different types of pairs, including adult-adult pairs and adult–child pairs, to further investigate potential shared sources. Among adult-adult pairs (*n* = 54), concentrations of benzophenone-3, BPA, BPF, and BPS were more strongly correlated among adult-adult pairs than among all pairs, whereas 2,5-dichlorophenol concentrations were less strongly correlated (tau = 0.32) than among all pairs (tau = 0.46) (Fig. S1). Like 2,5-dichlorophenol, propylparaben was less strongly correlated between adult–adult pairs (tau = 0.064) compared to all pairs (tau = 0.19). Among adult–child pairs (*n* = 26), benzophenone-3 was the most strongly correlated (tau = 0.39) followed by propylparaben (tau = 0.34) (Fig. S2). The correlation of propylparaben, a common preservative in personal care products, between adult–child pairs but not between adult-adult pairs may reflect important common sources between a child and a parent.

### The similarity of exposure-related behaviors

Self-reported exposure-related behaviors can help pinpoint important sources of exposure if they are highly correlated with urine concentrations. Similarly, the behaviors of other household members may influence participant exposures. In this study, we sought to quantify these influences as well as the residual contribution of a shared household to urine concentrations. Pair-wise evaluation of behaviors also provides context for a more comprehensive Bayesian hierarchical model that accounts for product use by multiple other participants in the same home.

**Fig. 2:**
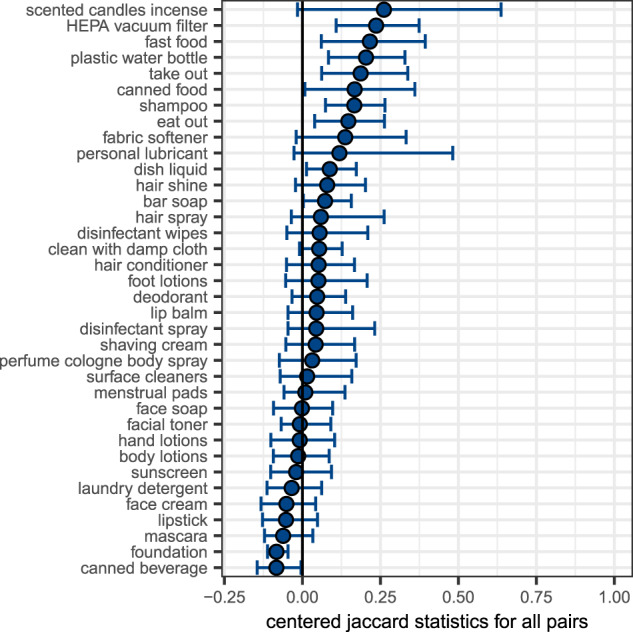
Similarity between self-reported behaviors. Similarity measured using centered Jaccard coefficient with bootstrapped 95% confidence intervals. Behaviors sorted by the Jaccard coefficient and exclude behaviors infrequently used or used by almost all participants (<10 users or <10 non-users). Participants were asked, “In the last 24 h, did you use this product [or do this activity]?”.

Several self-reported behaviors were similar between pair-members, including the use of HEPA vacuum cleaner in the home, drinking from a plastic water bottle, and eating canned food and take-out food in the last 24 h (Fig. 2). The size of the confidence intervals reflects the number of participants doing that behavior, with narrower confidence intervals indicating more common behaviors. For example, of the 65 participants who reported a HEPA vacuum cleaner being used in their home, 19 pairs (38 participants) reported use of a HEPA vacuum cleaner, with presumably the remaining pairs not realizing the type of vacuum being used. While eating fast food was reported by only 33 participants, 8 pairs (16 participants) both reported eating fast food. A third of all pairs reported both drinking out of a plastic water bottle whereas another third reported neither drinking out of a plastic water bottle. The personal care products that are most similar—shampoo, hand soap, and bar soap—are products that most participants are using. The similarity between pair members results from the use of both frequently used and infrequently used products, with certainty dependent on the number of participants reporting that behavior.

Dissimilar behaviors include make-up use (e.g., foundation, mascara) and drinking a canned beverage in the last 24 h. The majority of pairs are adult female and male pairs so we would expect make-up use to be discordant. Drinking a canned beverage was fairly common, with 46 participants reporting this behavior; however, only 6 participants had pair members also report this behavior.

### Contribution of personal behaviors, other household member behaviors, and sharing a home to biomonitored concentrations

We developed a Bayesian hierarchical model to more comprehensively evaluate the contribution of the personal behaviors, behaviors of others in the home, and the shared home environment. Both the pairwise correlation analysis of biomonitored concentrations and the pairwise intersection of behaviors suggest that other participants living in the same home share exposure sources, including exposure-related behaviors.

**Fig. 3:**
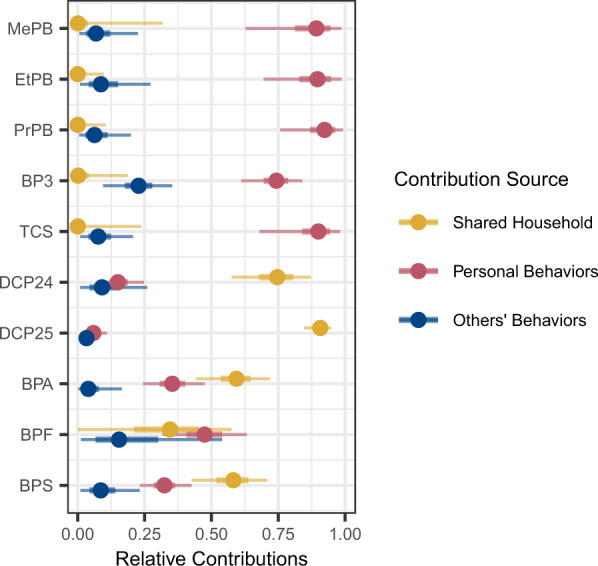
Relative contribution of personal behaviors, behaviors of others in the home, and remaining shared home exposure to measured urine concentration (total contribution is the sum of all three). Median estimates (dot), 50% credible interval (thicker line), and 90% credible interval (thinner line) are shown.

Personal behaviors contribute substantially to paraben, benzophenone-3, and triclosan concentrations (Fig. 3). For all three parabens, the relative contribution of personal behaviors is >75% of the total contribution of all three sources. Thus, most of the biomonitored concentrations for these parabens were explained by personal behavior with very little contribution from others’ behavior or from the shared home. Benzophenone-3 concentrations were influenced by personal behaviors and behaviors of others in the home, and the shared home had little influence. Benzophenone-3 concentrations were moderately correlated between pair-members. Because the shared home contributes minimally to benzophenone-3 concentrations, the correlation between pair members sharing a home is likely driven by shared behaviors. For example, plastic water bottle use, which is associated with higher benzophenone-3 concentrations (Fig. S3), is concordant between pair members. Similar results were observed for triclosan. The substantial contribution of personal behaviors to concentrations suggests that exposure reduction that targets the use of specific products associated with the exposure or modifying the composition of those products is likely to be effective.

Although behaviors of others in the household did not contribute the most relative to other sources for any chemical, they did contribute at least some to all of the chemicals (Fig. 3). The largest relative contribution was for benzophenone-3, where behaviors of others contributed ~20% to the total contribution (personal behaviors, behaviors of others, and the shared household). Benzophenone-3 is used in many products that are applied to the skin (e.g., sunscreens, lotions) so it is possible that a housemate may be exposed through direct contact, contact with contaminated surfaces, or contaminated indoor air or dust resulting from product use by others in the home. The small non-zero contribution by shared home may reflect building materials or ambient levels and may also reflect the contribution of personal and others’ product use behaviors that we did not ask about.

The shared household contribution includes sources that are shared among household members, such as building materials, furnishings, and drinking water, and also behaviors not accounted for in our survey. The shared household contributed substantially to the concentrations of the dichlorophenols, BPA, and BPS (Fig. 3). 2,5-Dichlorophenol, which was highly correlated among pair-members (Fig. 1), had the highest contribution from the shared household. This is consistent with the known sources of 2,5-dichlorophenol, which is a metabolite of a disinfectant and pesticide used in mothballs and deodorizers and also a potential drinking water contaminant, all sources that are likely to affect all household members by affecting the ambient home environment [14, 16, 40–42]. 2,4-Dichlorophenol is a minor degradation product of triclosan, a metabolite of the herbicide 2,4-D and the wood preservative pentachlorophenol, and a potential drinking water contaminant [13, 15, 42]. Urine levels of 2,4-dichlorophenol are modestly correlated among pair-members (Fig. 1) and key exposure sources are uncertain since levels were not associated with the use of the products we asked about, except for an association with triclosan use in some participants [34]. BPA and BPS levels were modestly correlated among pair members (Fig. 1), and the shared home is identified as relatively more important than personal or others’ product use (Fig. 3). While these chemicals may be found in building materials, they are also used in consumer goods used by an individual, so other behaviors not asked about may be important for bisphenols such as BPA and BPS. In our analysis of the entire DMAK cohort, we did not find positive associations between the anticipated sources of bisphenols and measured urine concentrations [34]. Additional research on the influential sources of these chemicals is needed.

### Strengths and limitations

Using data from our crowdsourced biomonitoring study, we used a novel statistical approach to estimate the contribution of personal behaviors, behaviors of others in the home, and the shared home environment on measured urine concentrations of phenolic consumer product chemicals. Our results help pinpoint exposure sources and also inform intervention strategies. While our approach was novel and we leveraged an opportunity to address a specific research question related to the contribution of the shared home on biomonitored concentrations, there are some limitations, which are mostly a result of the fact that the underlying study was not designed specifically for this analysis. First, we did not explicitly ask participants if there was another participant sharing their home. Instead, we assumed a shared home if participants used the same shipping address. We note, however, that 17 households (of the 82 shared homes in this analysis) joined the study specifically to investigate correlated exposures among individuals in their household. Second, we did not ask for the exact date of urine collection and had to estimate this based on the dates when the samples were returned; and then we used these dates to subset our data to participants who returned their kits within 4 weeks of another participant in their shared home. We selected 4 weeks since we expected behaviors and urine concentrations to be relatively stable over this short time period. We tested this assumption by comparing results from the pairwise concentration correlation analysis, similarity of exposure-related behaviors analysis, and contribution analysis for participants who returned their urine samples within 4 weeks of another participant in their home to participants who returned their urine samples within 1 week of another participant (see Supplementary Information, Fig. S4). We note that the majority of pairs returned kits within 1 week of each other (over 80% of pairs have concentration data from samples returned within 1 week). Overall, the results were similar using the two time periods so we are confident that participants’ samples returned within 4 weeks reflect near-simultaneous home product use, behaviors, and home conditions. Having multiple (mostly two but up to six) participants per home is both a strength and limitation of our analysis. We investigated the effect of clustering on our pairwise analyses by eliminating multiple pairs from within the same household and found little impact on the pairwise correlation estimates. Clustering is not an issue in our contribution analysis since the Bayesian model accounts for multiple participants within a home.

## Conclusion

Urine concentrations for ten phenolic hormonally-active consumer product chemicals are correlated among participants living in the same home. These correlations can help pinpoint important sources of exposure for reformulation or reduction in use. While personal product use contributes the most to urinary concentrations of parabens, benzophenone-3, and triclosan, the shared household contributes the most to urinary concentrations of 2,5-dichlorophenol, 2,4-dichlorophenol, BPA, and BPS. Shared household exposures include building materials, furnishings, and other consumer products not asked about in this study. Future studies should try to identify shared sources of exposure within the home environment including building materials and furnishings and other specific personal behaviors associated with exposure. Understanding shared sources of exposure is important so that intervention strategies can be developed that will reduce exposures, not just for an individual but rather everyone in the home. This is especially important when we consider actions that can be taken to improve housing conditions, limit exposures, and improve the health of susceptible or vulnerable individuals, including children.

## Supplementary information

Supplementary information
