# Supplementary material for: Influence of living in the same home on biomonitored levels of consumer product chemicals
Source: J Expo Sci Environ Epidemiol. 2021 Jul 13;32(6):885–91. doi: 10.1038/s41370-021-00368-8 (PMC9731902; doi:10.1038/s41370-021-00368-8)
Supplement: Supplementary file 1 — Supplementary information [file 41370_2021_368_MOESM1_ESM.docx]

**SUPPLEMENTARY INFORMATION FOR:**

**Influence of living in the same home on biomonitoring levels of consumer product chemicals in the Detox Me Action Kit crowdsourced study**

Robin E. Dodson,^1^ R. Woodrow Setzer,^2^ John D. Spengler,^3^ Ruthann A. Rudel,^1^ Jose Guillermo Cedeño Laurent^2^

*^1^Silent Spring Institute, Newton MA USA; ^2^Retired, Chapel Hill, NC USA; ^3^Harvard T.H. Chan School of Public Health, Boston, MA USA*

**Contents:**

*A priori* exposure-related behaviors

Pair-wise correlation estimates for adult-adult pairs and adult-child pairs

Effect estimates for personal behaviors and total product use by other household members

Sensitivity analysis of time period selection - Analysis of time-restricted (samples returned within one week of each other) data set

Bayesian model specifications

Table S1. Exposure-related behaviors (24 hour recall) selected a priori as relevant predictors of exposure by chemical(s)

| **Exposure-related behavior** | **Relevant Chemical(s)** | |
| --- | --- | --- |
| canned beverage | bisphenols | |
| canned food | bisphenols | |
| eat out | bisphenols | |
| fast food | bisphenols | |
| plastic container | bisphenols, benzophenone-3 | |
| plastic water bottle | bisphenols, benzophenone-3 | |
| take out | bisphenols | |
| body lotions | parabens, benzophenone-3 | |
| deodorant | parabens, triclosan | |
| face cream | parabens, benzophenone-3 | |
| face soap | parabens, triclosan | |
| facial masks | parabens, triclosan | |
| foot lotions | parabens | |
| foundation | parabens, benzophenone-3 | |
| hair conditioner | parabens | |
| hair shine | parabens | |
| hair spray | parabens | |
| hand lotions | parabens, benzophenone-3 | |
| hand soap | parabens, triclosan | |
| lip balm | parabens, benzophenone-3 | |
| lipstick | parabens, benzophenone-3 | |
| mascara | parabens | |
| personal lubricant | parabens | |
| shampoo | parabens | |
| shaving cream | parabens | |
| sunscreen | parabens, benzophenone-3 | |
| toner | parabens | |
| bar soap | triclosan | |
| dish liquid | triclosan | |
| disinfectant spray | triclosan | |
| disinfectant wipes | Triclosan | |
| surface cleaners | triclosan, dichlorophenols | |
| toothpaste | Triclosan | |
| moth balls | dichlorophenols | |
| tampons | dichlorophenols | |
| toilet bowl cleaner | dichlorophenols | |
| toilet bowl deodorizers | dichlorophenols |  |


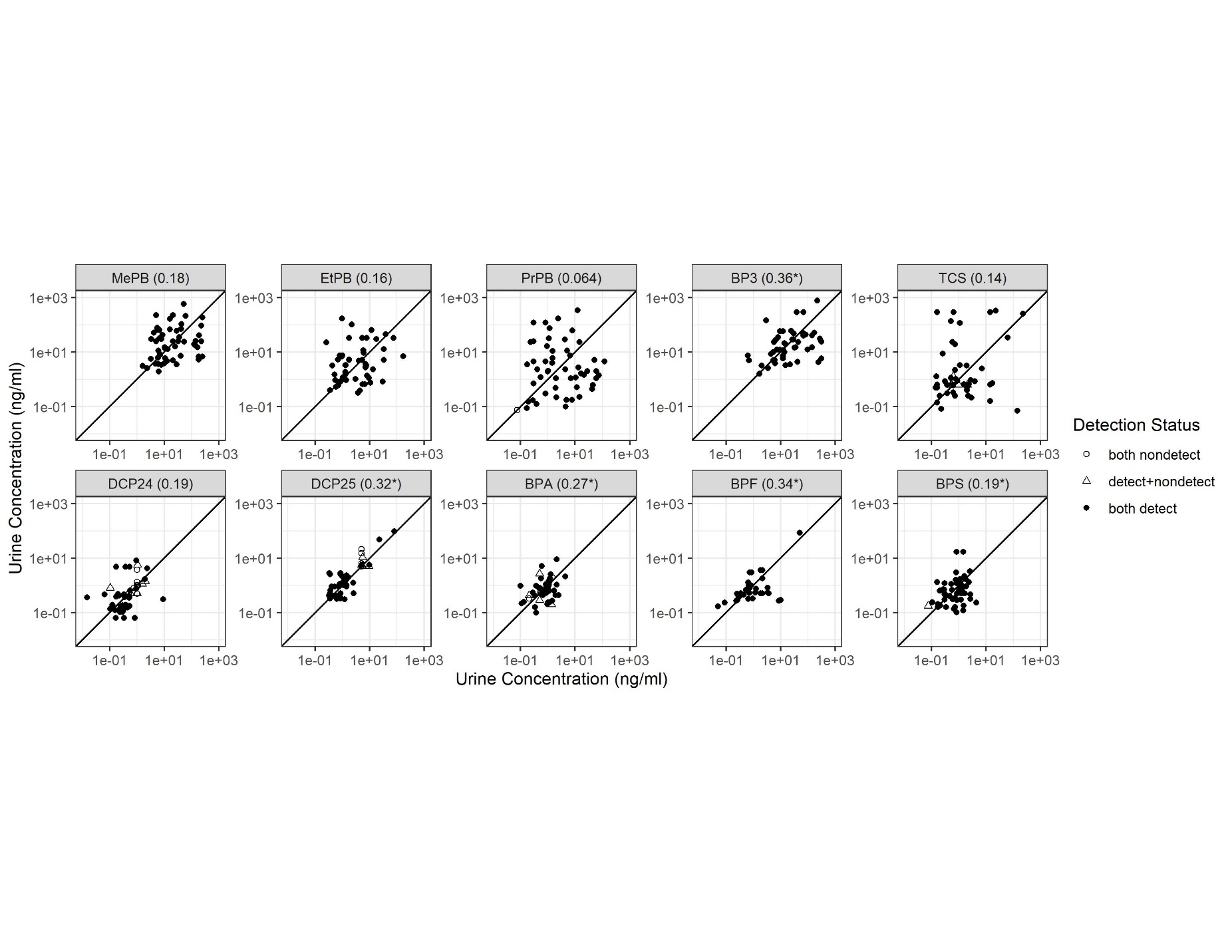


## Figure S1. Scatterplots of measured urinary concentrations for adult-adult pairs (1 pair member on x-axis and 1 pair member on y-axis). Note logarithmic scale. 1:1 line shown. Point shapes indicate whether both values were above the MRL, one value was above and one value was below the MRL, or both were below the MRL. Values below the MRL are plotted at the MRL. Kendall’s tau beta correlation estimates in parentheses. Asterisks indicates significant correlation (p<0.05).

##
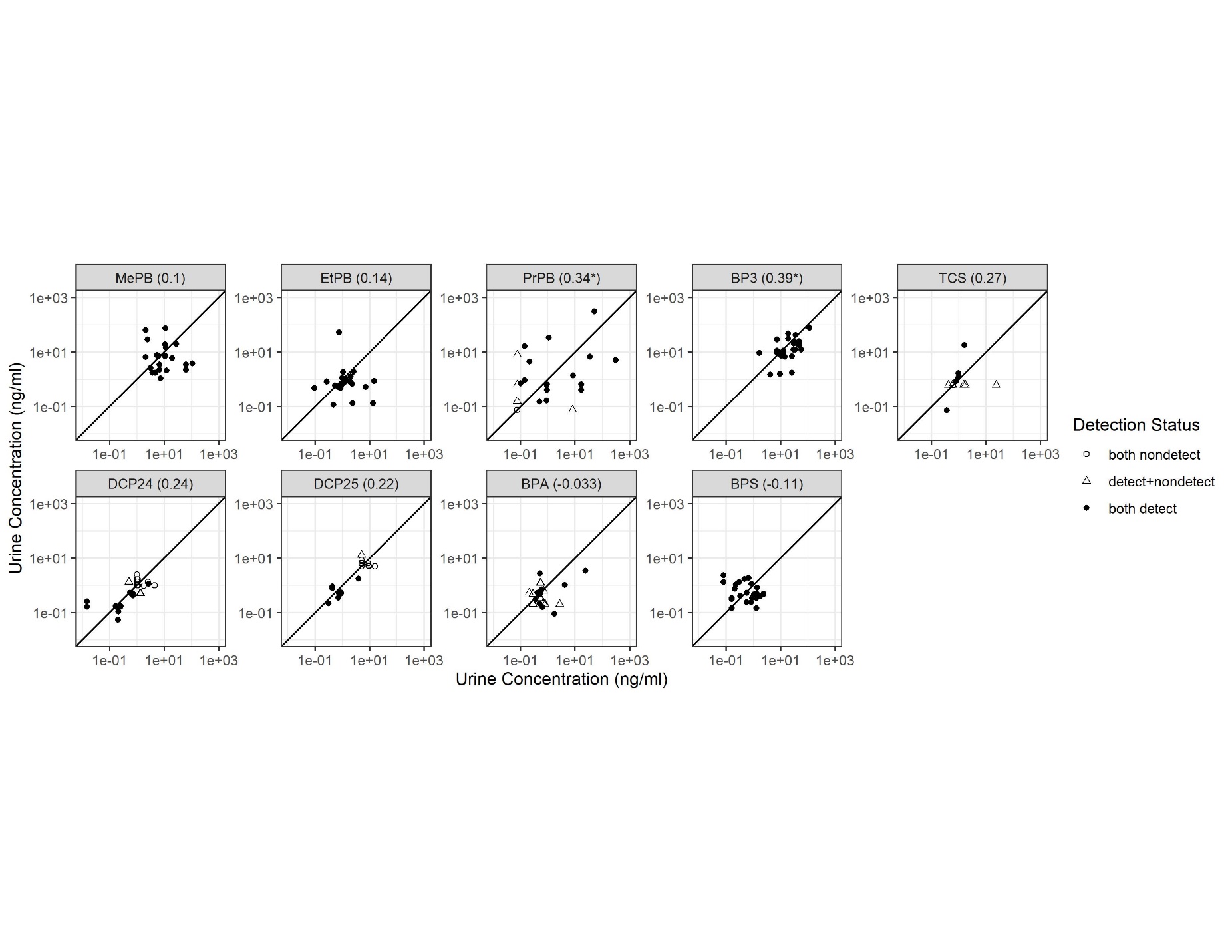


## Figure S2. Scatterplots of measured urinary concentrations for adult-child pairs (1 pair member on x-axis and 1 pair member on y-axis). Note logarithmic scale. 1:1 line shown. Point shapes indicate whether both values were above the MRL, one value was above and one value was below the MRL, or both were below the MRL. Values below the MRL are plotted at the MRL. Kendall’s tau beta correlation estimates in parentheses. Asterisks indicates significant correlation (p<0.05). BPF not shown because of insufficient number of simultaneous detects.


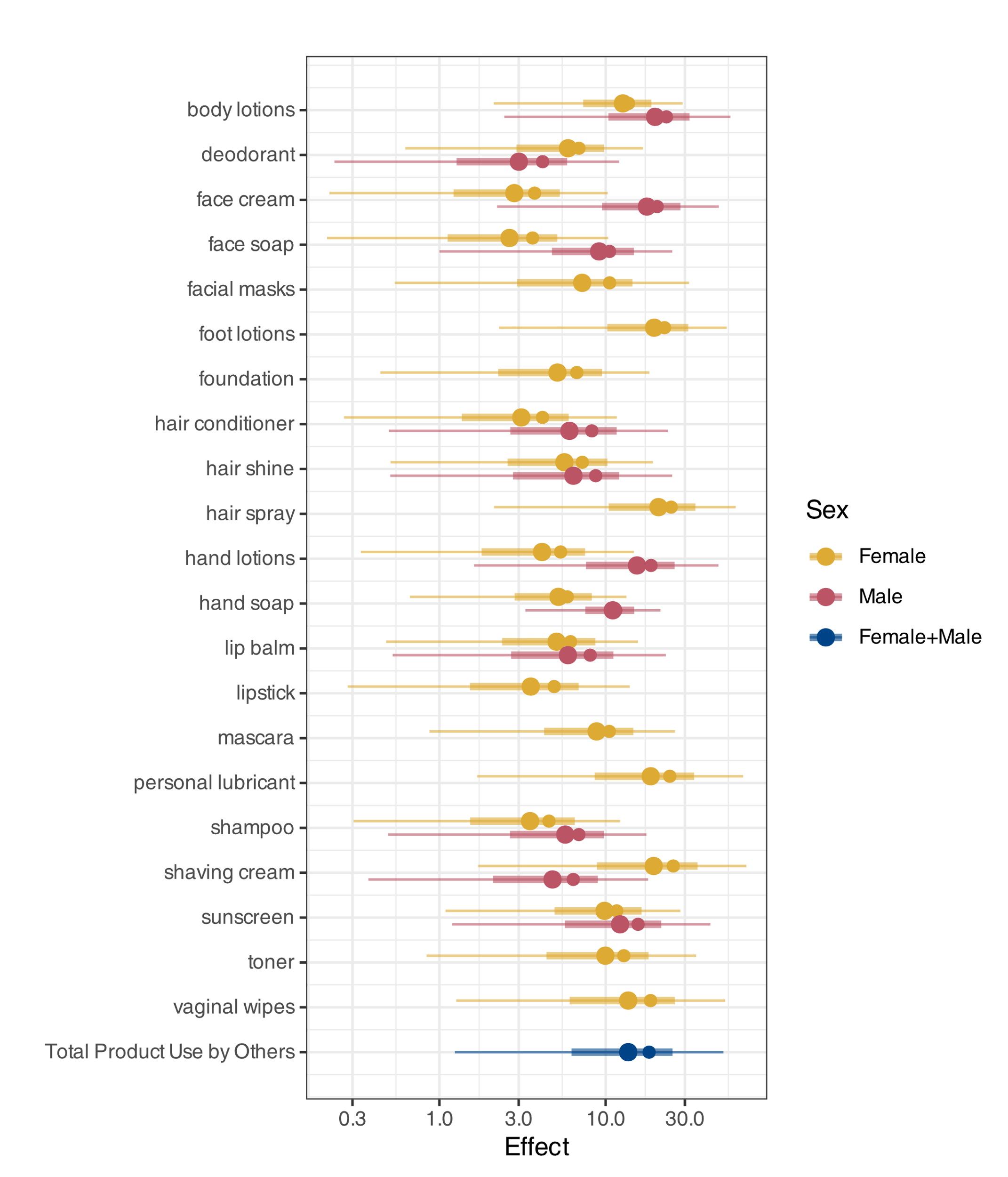


Figure S3a. Associations between specific product use and total product use by others in the household and measured urine concentrations of **methyl paraben** for females (yellow or light gray), males (red or medium gray), and both females and males (blue or dark gray). Median (large point), mean (small point), 50% credible interval (wide bar), and 90% credible interval (narrow bar) estimates of population posterior distribution of effect estimates for personal behaviors and total product use by others in the household and measured urine concentrations.


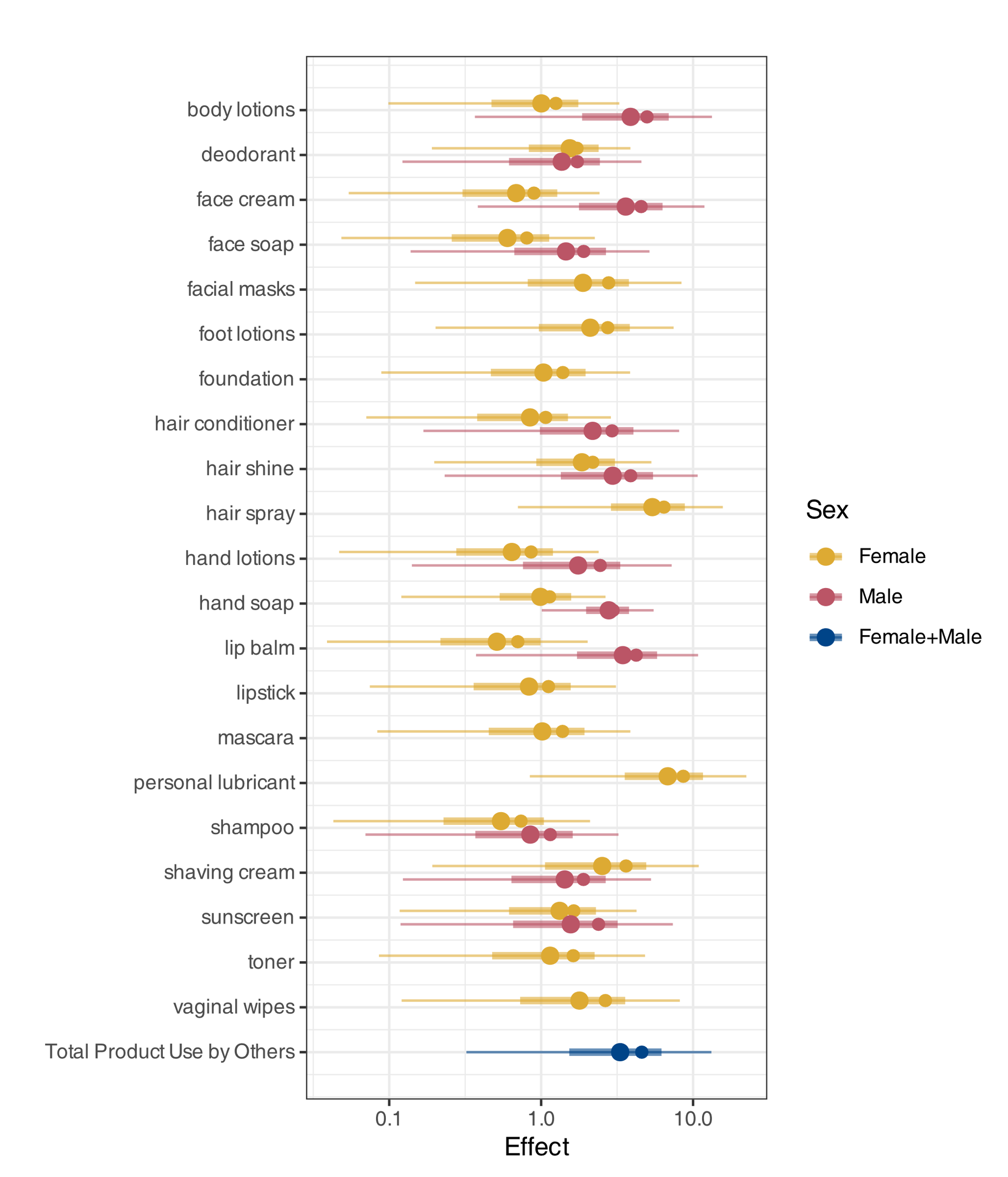


Figure S3b. Associations between specific product use and total product use by others in the household and measured urine concentrations of **ethyl paraben** for females (yellow or light gray), males (red or medium gray), and both females and males (blue or dark gray). Median (large point), mean (small point), 50% credible interval (wide bar), and 90% credible interval (narrow bar) estimates of population posterior distribution of effect estimates for personal behaviors and total product use by others in the household and measured urine concentrations.


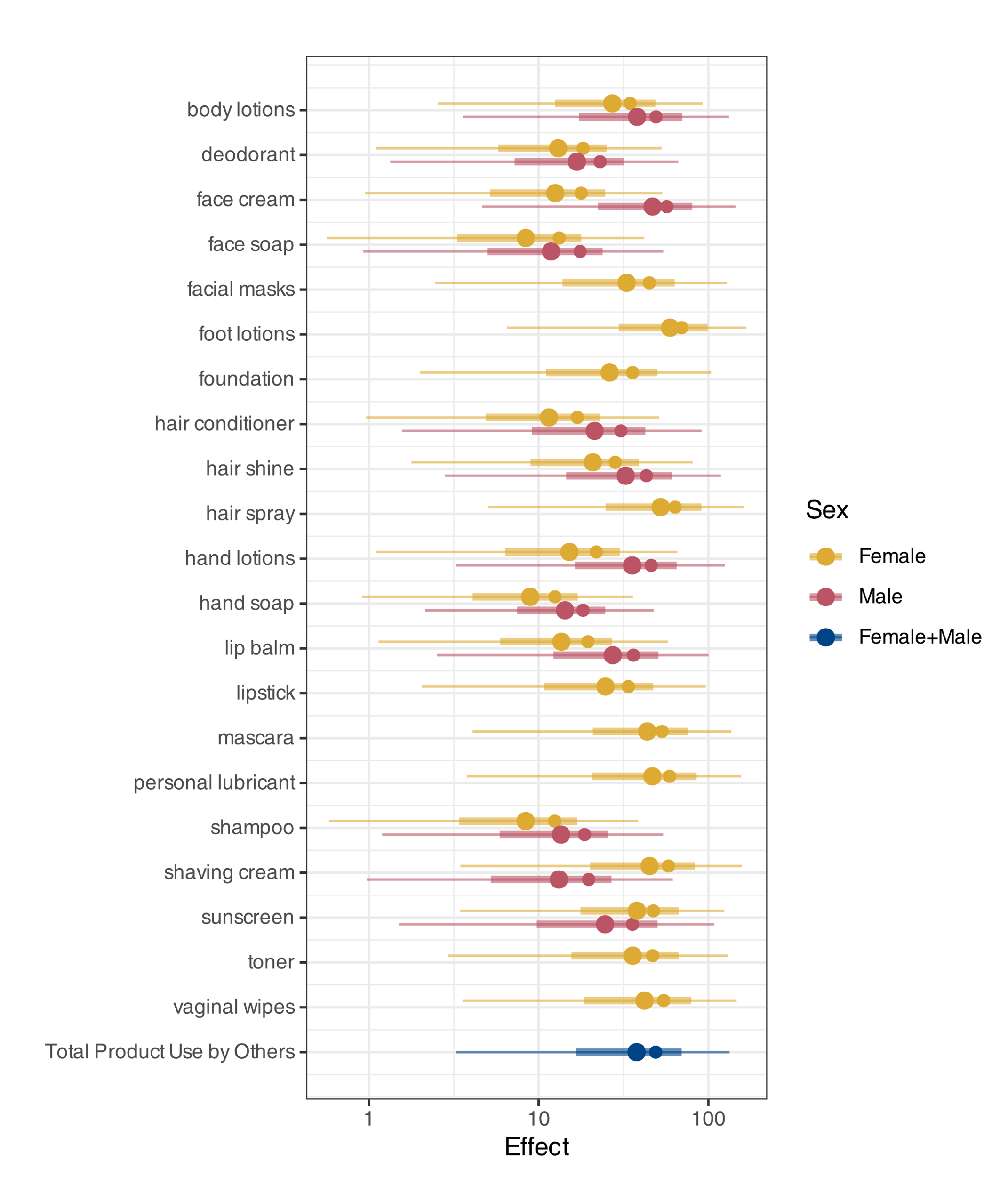


Figure S3c. Associations between specific product use and total product use by others in the household and measured urine concentrations of **propyl paraben** for females (yellow or light gray), males (red or medium gray), and both females and males (blue or dark gray). Median (large point), mean (small point), 50% credible interval (wide bar), and 90% credible interval (narrow bar) estimates of population posterior distribution of effect estimates for personal behaviors and total product use by others in the household and measured urine concentrations.


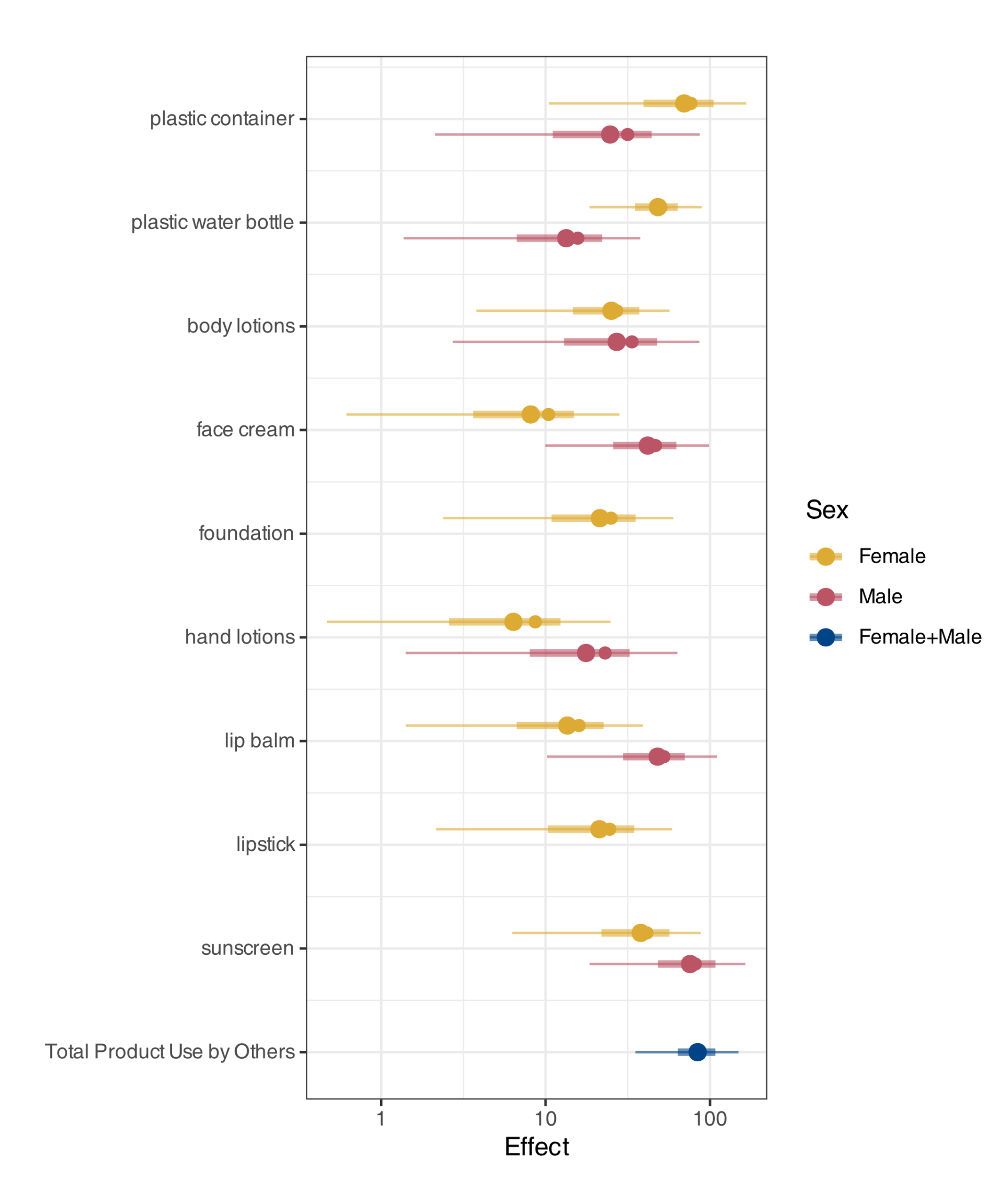


Figure S3d. Associations between specific product use and total product use by others in the household and measured urine concentrations of **benzophenone-3** for females (yellow or light gray), males (red or medium gray), and both females and males (blue or dark gray). Median (large point), mean (small point), 50% credible interval (wide bar), and 90% credible interval (narrow bar) estimates of population posterior distribution of effect estimates for personal behaviors and total product use by others in the household and measured urine concentrations.


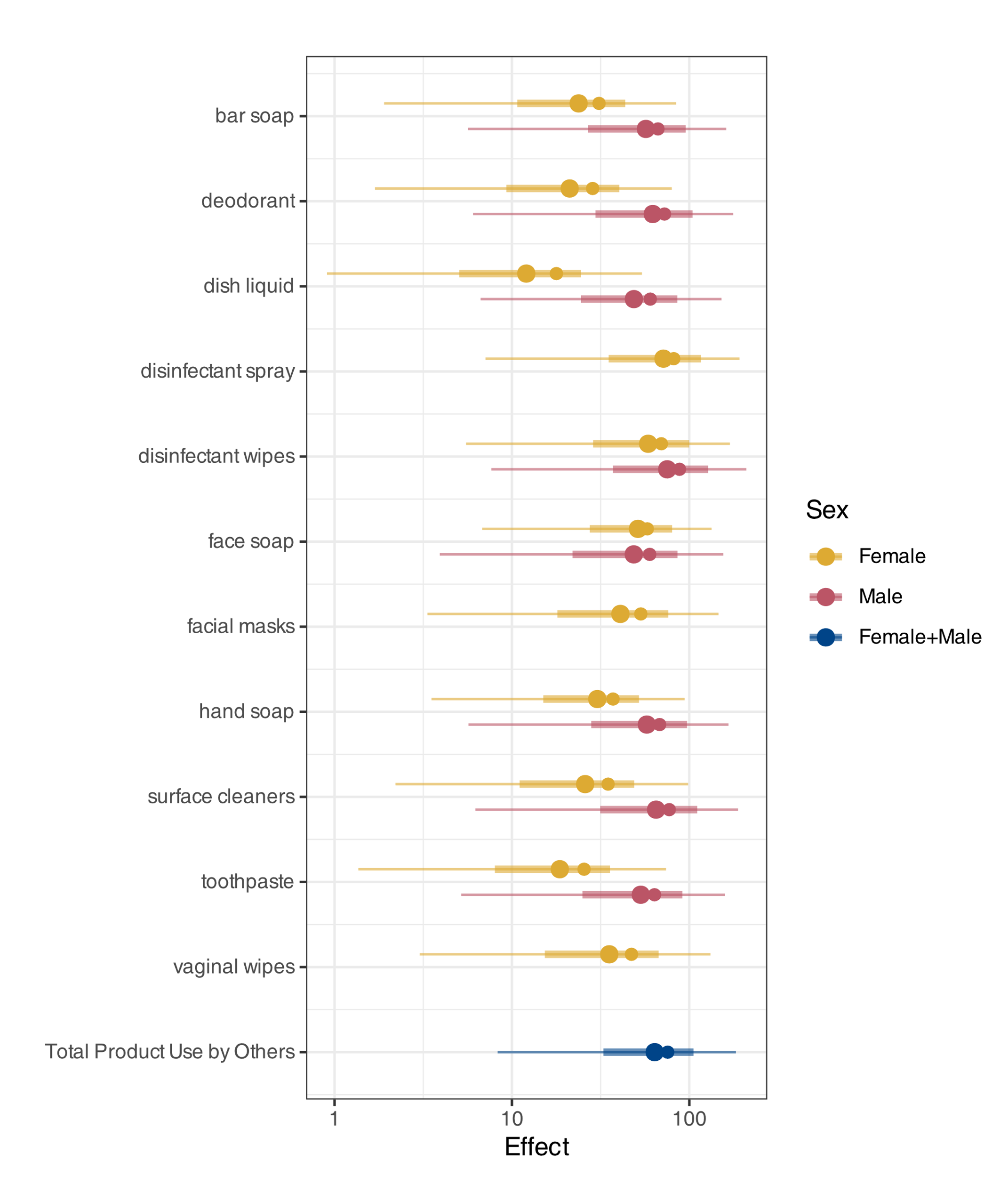


Figure S3e. Associations between specific product use and total product use by others in the household and measured urine concentrations of **triclosan** for females (yellow or light gray), males (red or medium gray), and both females and males (blue or dark gray). Median (large point), mean (small point), 50% credible interval (wide bar), and 90% credible interval (narrow bar) estimates of population posterior distribution of effect estimates for personal behaviors and total product use by others in the household and measured urine concentrations.


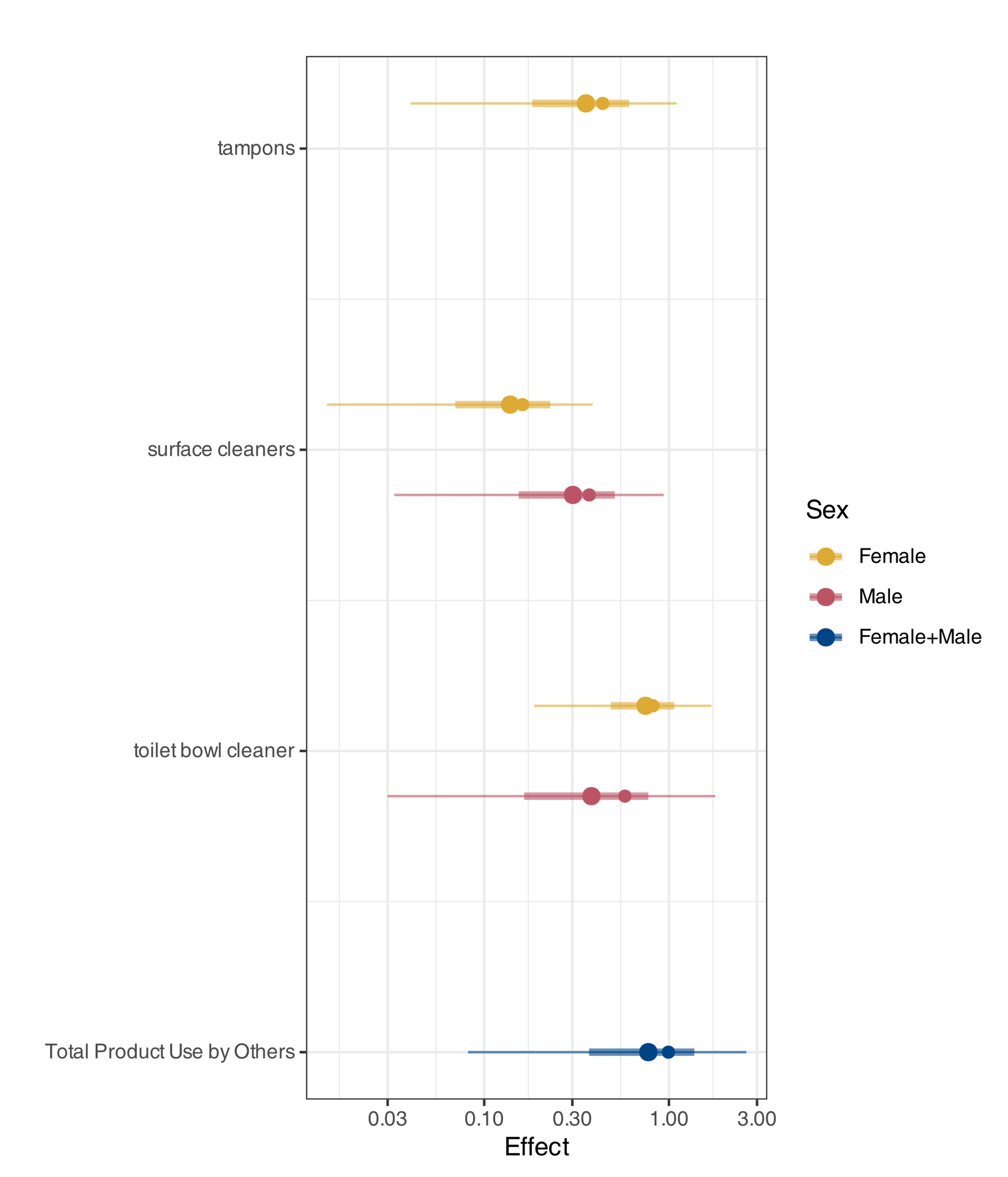


Figure S3f. Associations between specific product use and total product use by others in the household and measured urine concentrations of **2,4-dichlorophenol** for females (yellow or light gray), males (red or medium gray), and both females and males (blue or dark gray). Median (large point), mean (small point), 50% credible interval (wide bar), and 90% credible interval (narrow bar) estimates of population posterior distribution of effect estimates for personal behaviors and total product use by others in the household and measured urine concentrations.


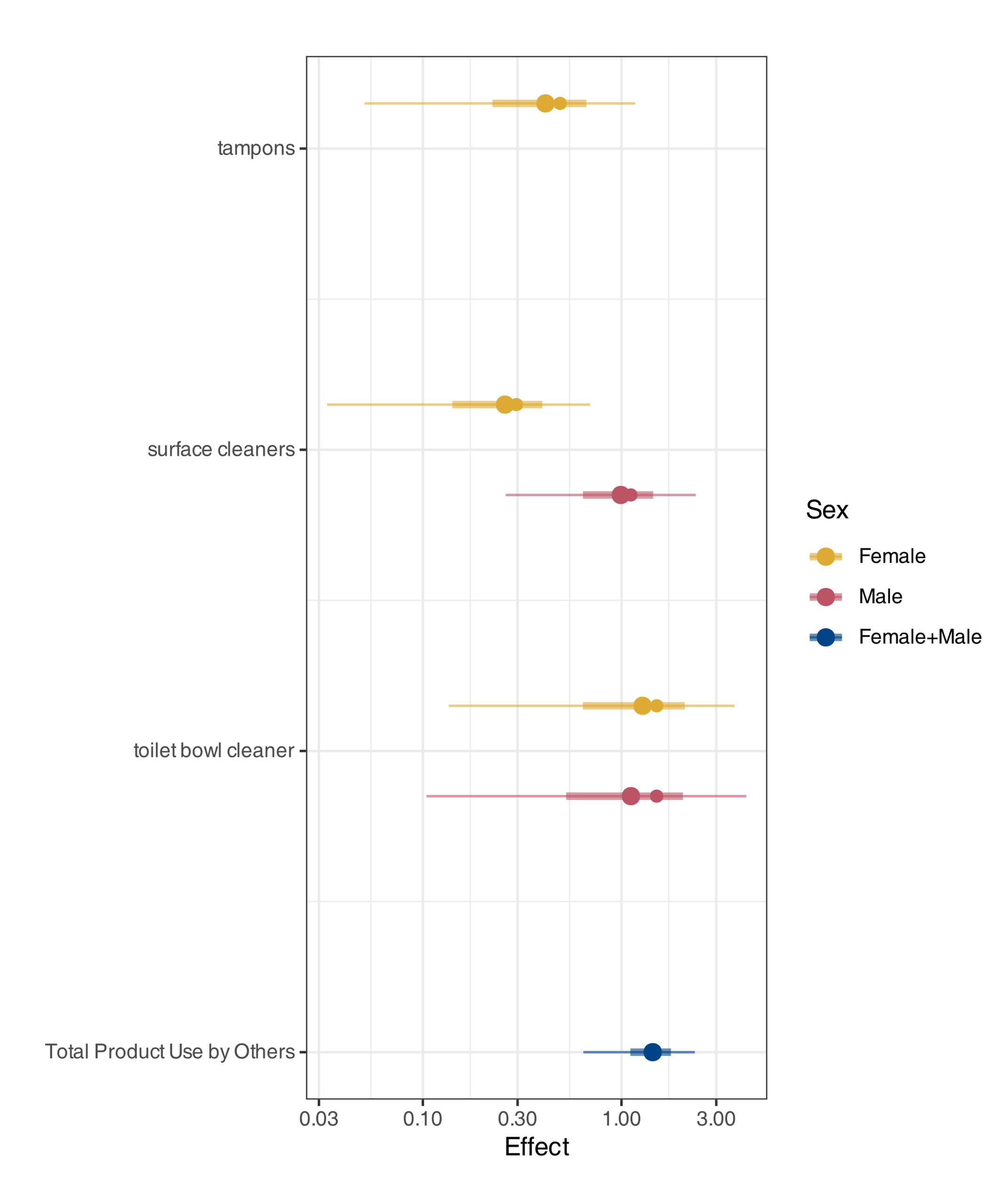


Figure S3g. Associations between specific product use and total product use by others in the household and measured urine concentrations of **2,5-dichlorophenol** for females (yellow or light gray), males (red or medium gray), and both females and males (blue or dark gray). Median (large point), mean (small point), 50% credible interval (wide bar), and 90% credible interval (narrow bar) estimates of population posterior distribution of effect estimates for personal behaviors and total product use by others in the household and measured urine concentrations.


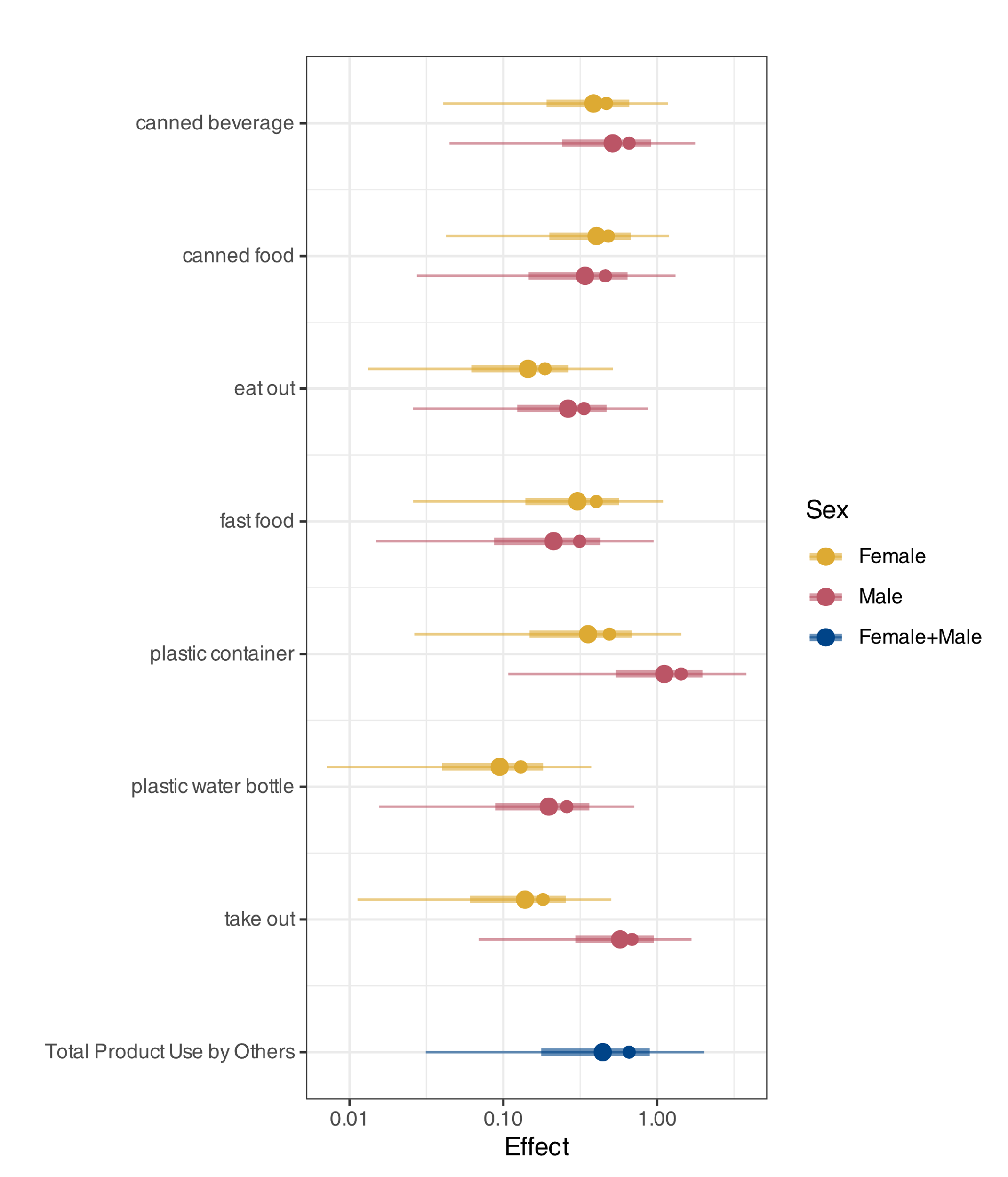


Figure S3h. Associations between specific product use and total product use by others in the household and measured urine concentrations of **bisphenol A** for females (yellow or light gray), males (red or medium gray), and both females and males (blue or dark gray). Median (large point), mean (small point), 50% credible interval (wide bar), and 90% credible interval (narrow bar) estimates of population posterior distribution of effect estimates for personal behaviors and total product use by others in the household and measured urine concentrations.


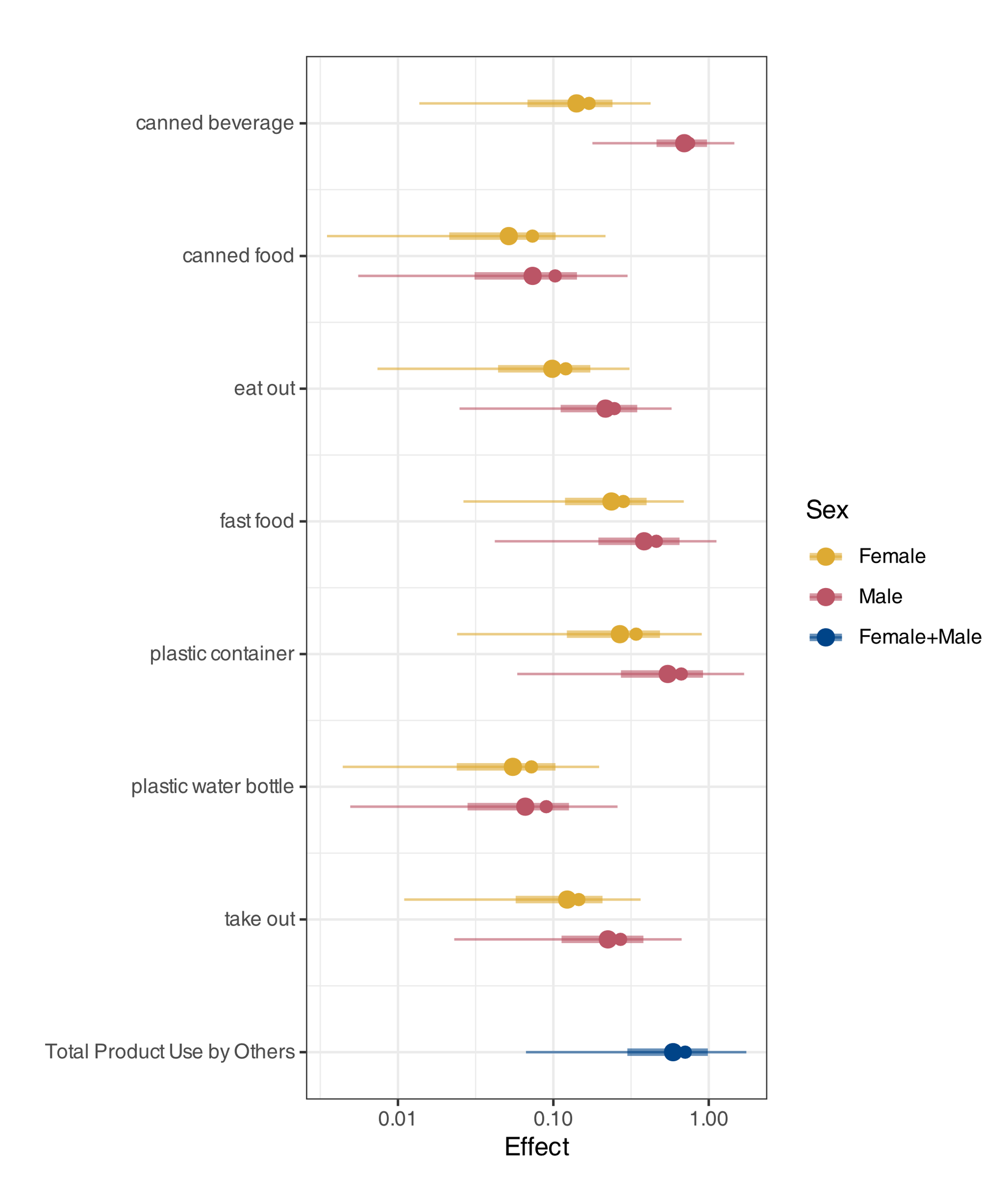


Figure S3i. Associations between specific product use and total product use by others in the household and measured urine concentrations of **bisphenol F** for females (yellow or light gray), males (red or medium gray), and both females and males (blue or dark gray). Median (large point), mean (small point), 50% credible interval (wide bar), and 90% credible interval (narrow bar) estimates of population posterior distribution of effect estimates for personal behaviors and total product use by others in the household and measured urine concentrations.


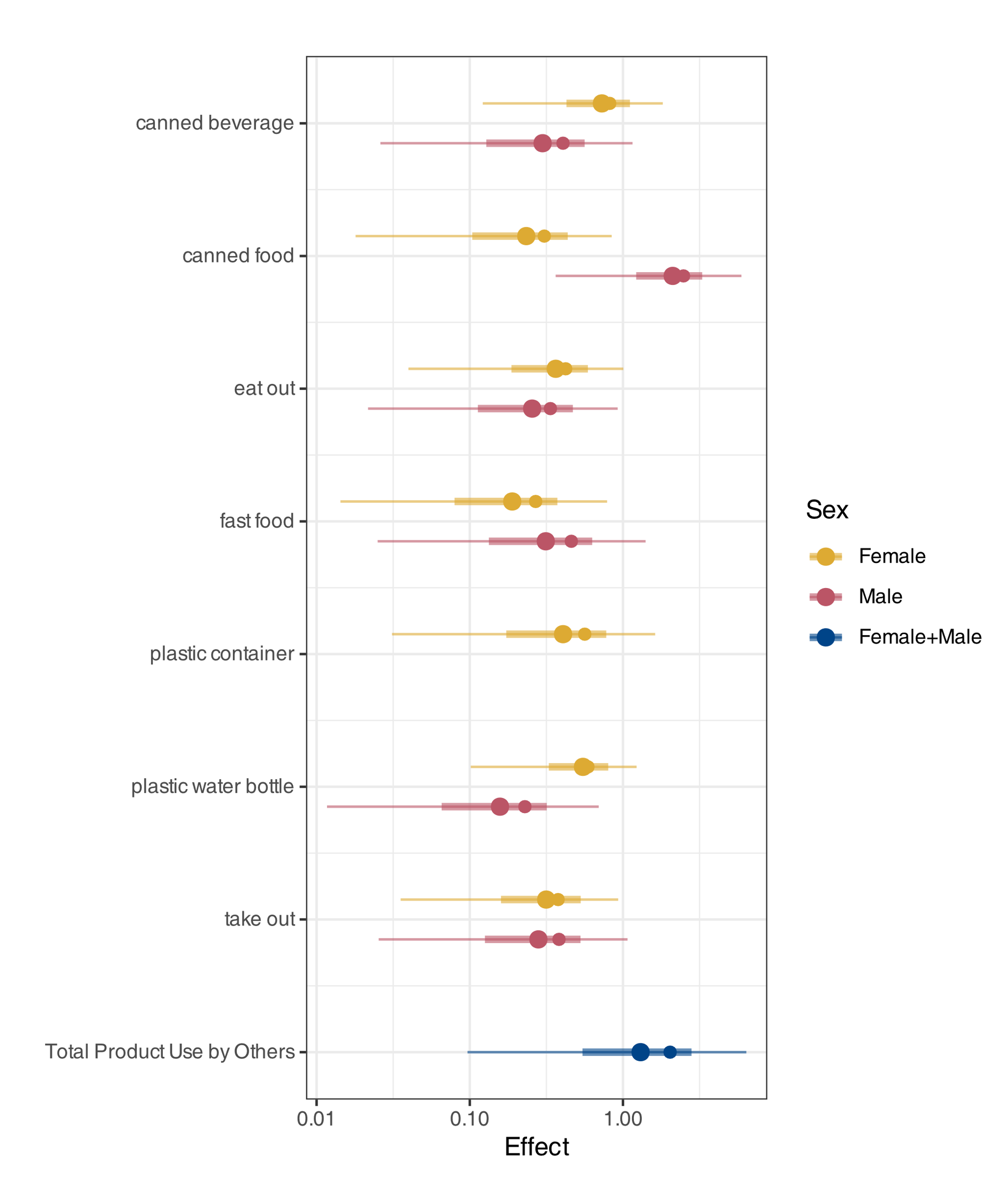


Figure S3j. Associations between specific product use and total product use by others in the household and measured urine concentrations of **bisphenol S** for females (yellow or light gray), males (red or medium gray), and both females and males (blue or dark gray). Median (large point), mean (small point), 50% credible interval (wide bar), and 90% credible interval (narrow bar) estimates of population posterior distribution of effect estimates for personal behaviors and total product use by others in the household and measured urine concentrations.


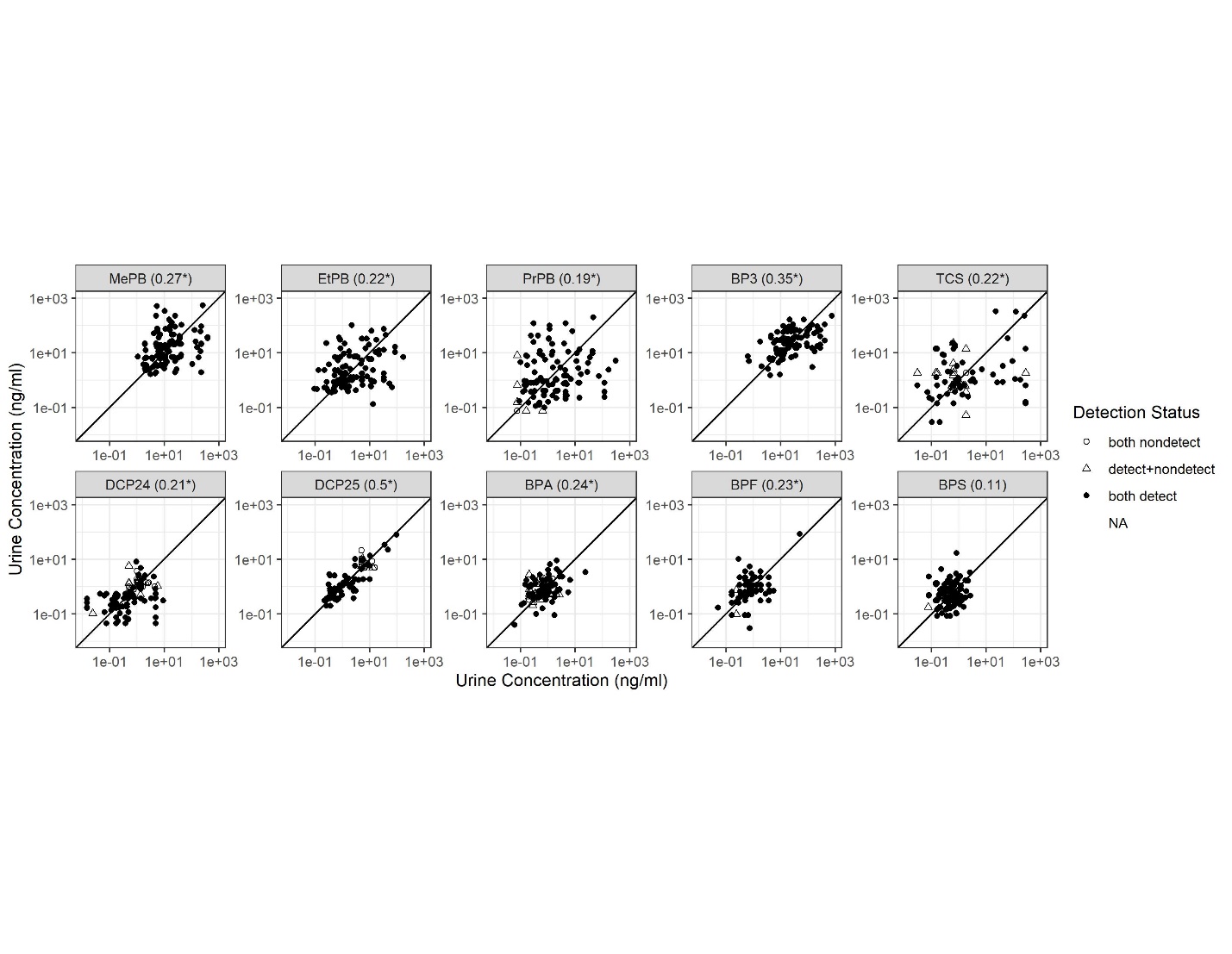


Figure S4a. Scatterplots of measured urinary concentrations for each pair (1 pair member on x-axis and 1 pair member on y-axis) that returned their samples **within one week of each other (n=111)**. Note logarithmic scale. 1:1 line shown. Point shapes indicate whether both values were above the MRL, one value was above and one value was below the MRL, or both were below the MRL. Values below the MRL are plotted at the MRL. Kendall’s tau beta correlation estimates in parentheses. Asterisks indicate significant correlation (p<0.05).


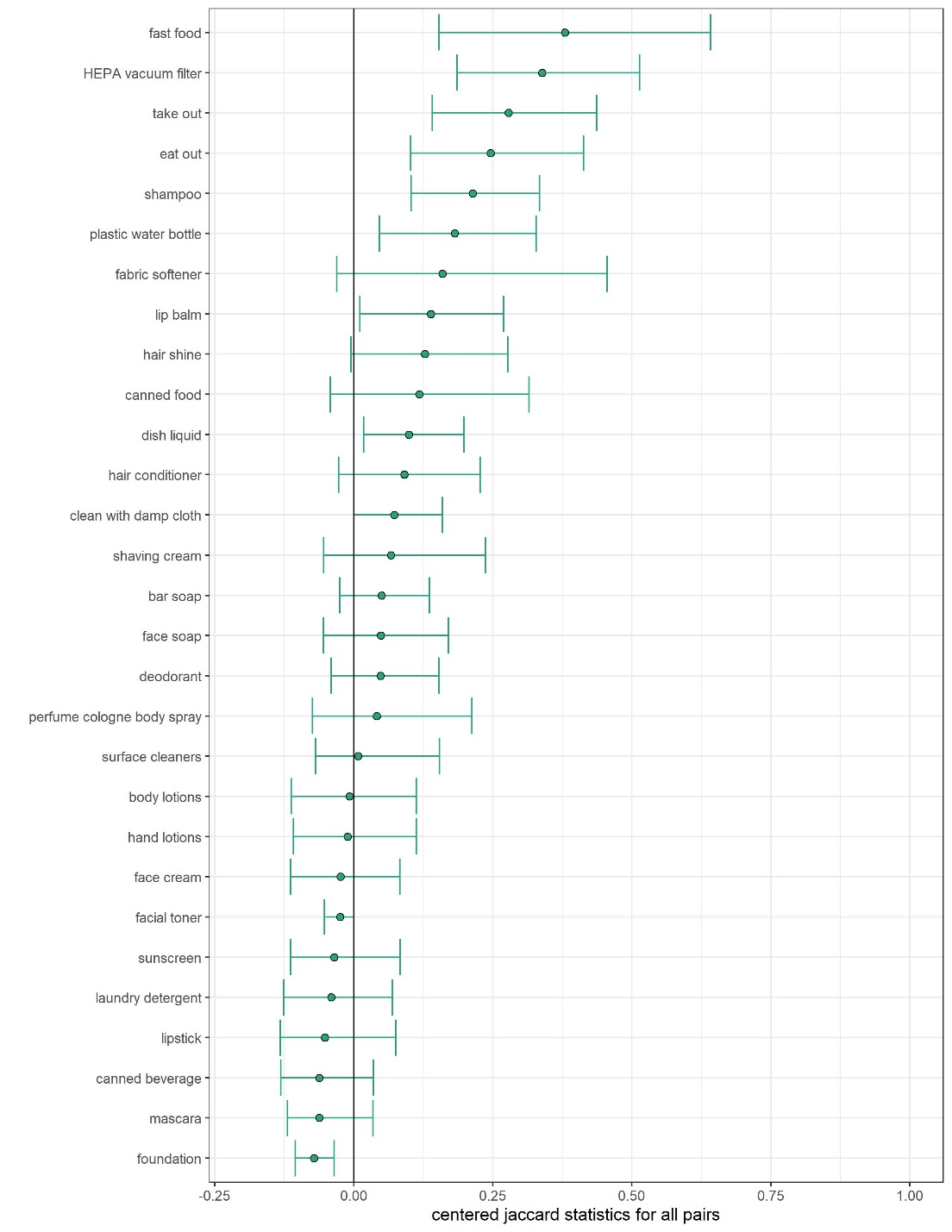


## Figure S4b. Similarity between self-reported behaviors **among pairs who returned their samples within one week of each other (n=87 pairs).** Similarity measured using centered Jaccard coefficient with bootstrapped 95% confidence intervals. Behaviors sorted by Jaccard coefficient and excludes behaviors infrequently used or used by almost all participants (<10 users or <10 non-users). Participants were asked, “In the last 24 hours, did you use this product [or do this activity]?”

We repeated the Bayesian analysis on a time-restricted dataset that included **only participants who returned their samples within one week** of at least one other participant from the same household. Estimates of ICC, the relative contributions of household, personal product use, and other household members' product use to total exposure were similar for both full and time-restricted datasets (Figure S4c). Additionally, estimates of the individual effects of product use were similar between the two datasets (S4d-e). Based on this analysis, we believe that the 4 week time period used in our analysis was adequate for our purposes.


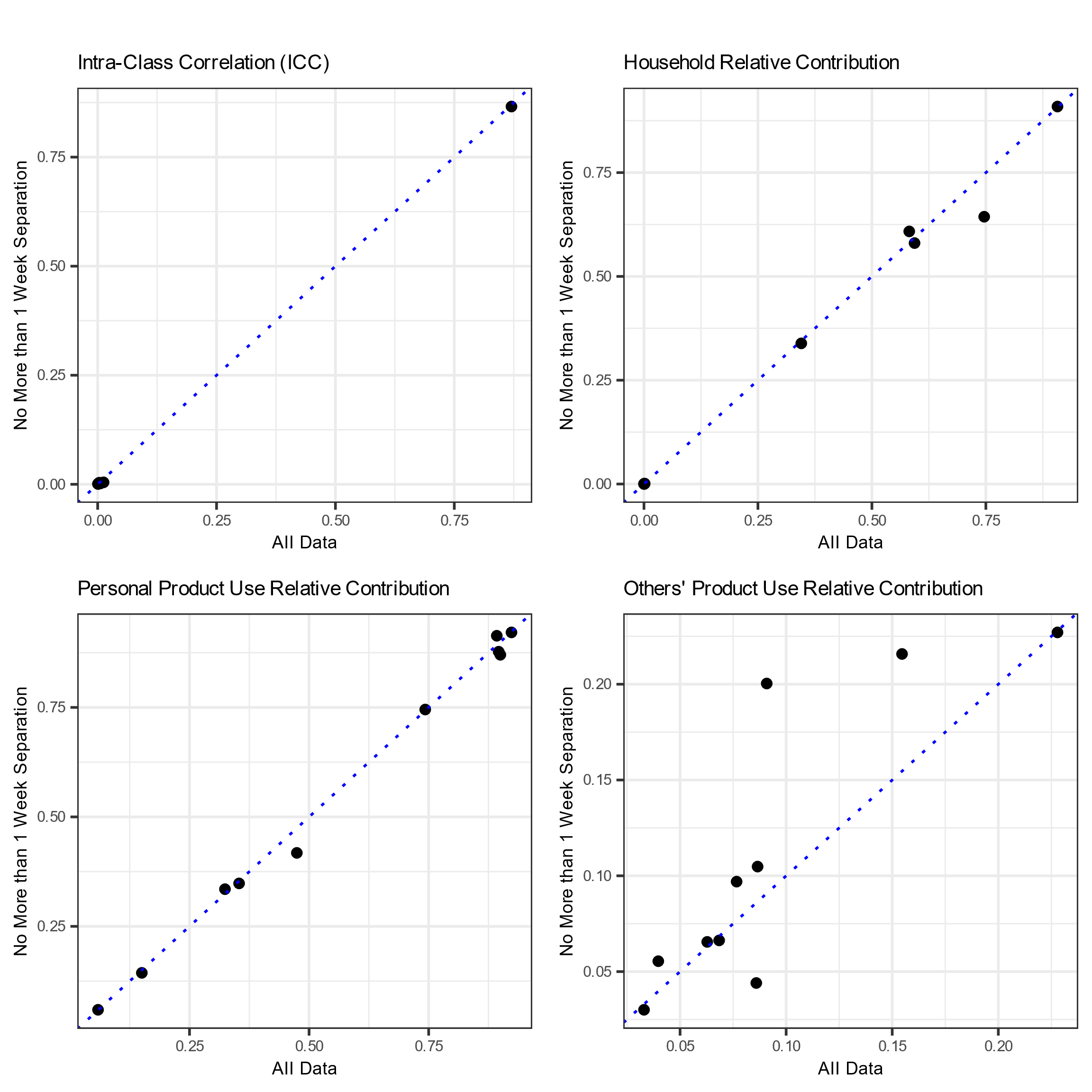


Figure S4c. Comparison of estimates relevant parameters from full dataset (abscissa) and the time-restricted dataset (ordinate). Perfect concordance would be indicated by all the points falling on the diagonal identity line. Top left is the intraclass correlation coefficient (ICC), top right the fractional contribution of household to total average exposure, bottom left, contribution of personal product use, and bottom right, total product use by other household members.


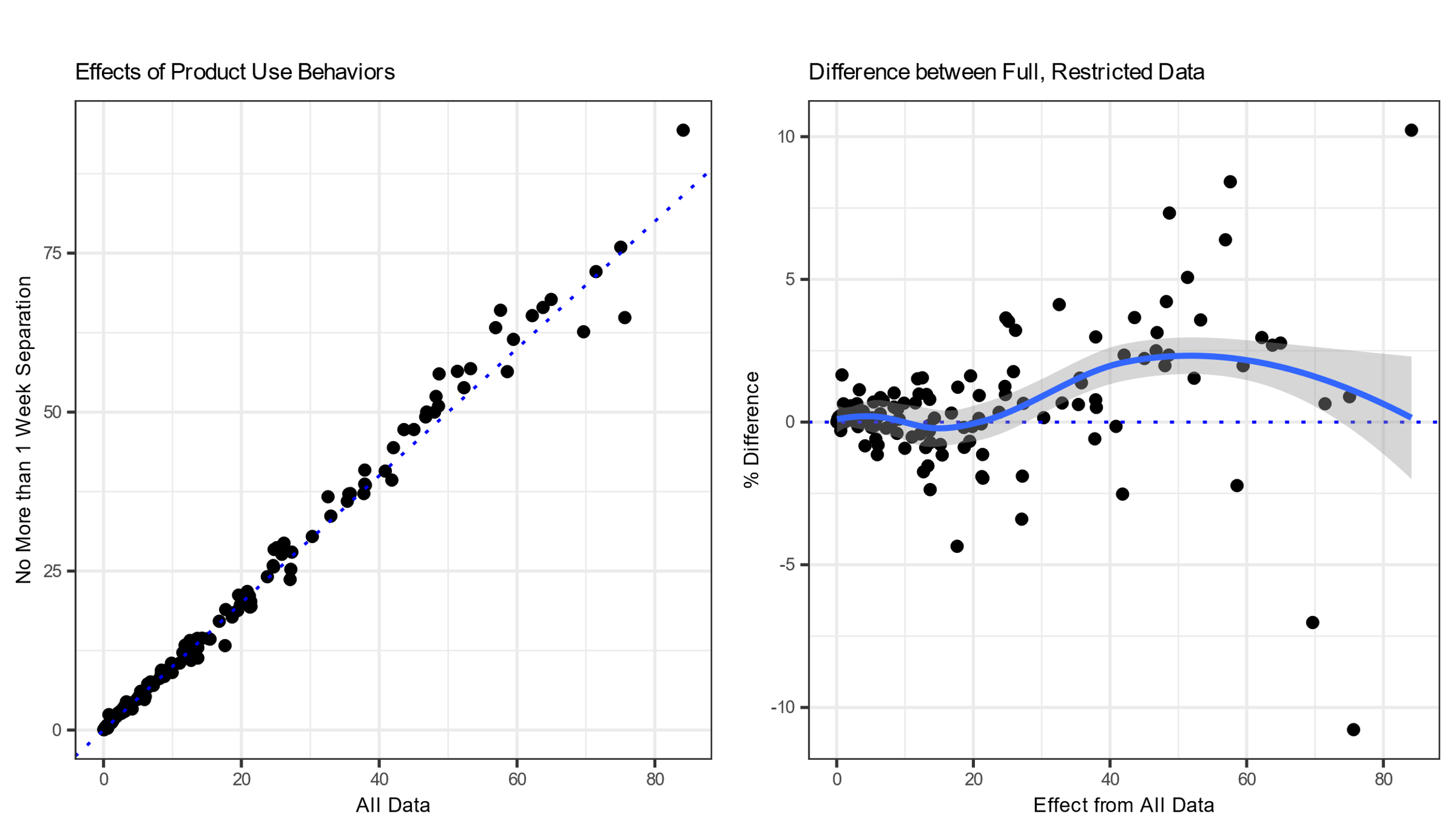


Figure S4d. Comparison of estimates of effects of product use behaviors between full dataset (abscissa) and the time-restricted dataset (ordinate), on the left. On the right, the difference between the two estimates is plotted against the estimate from the full dataset. The curve is a loess smooth through the data.**
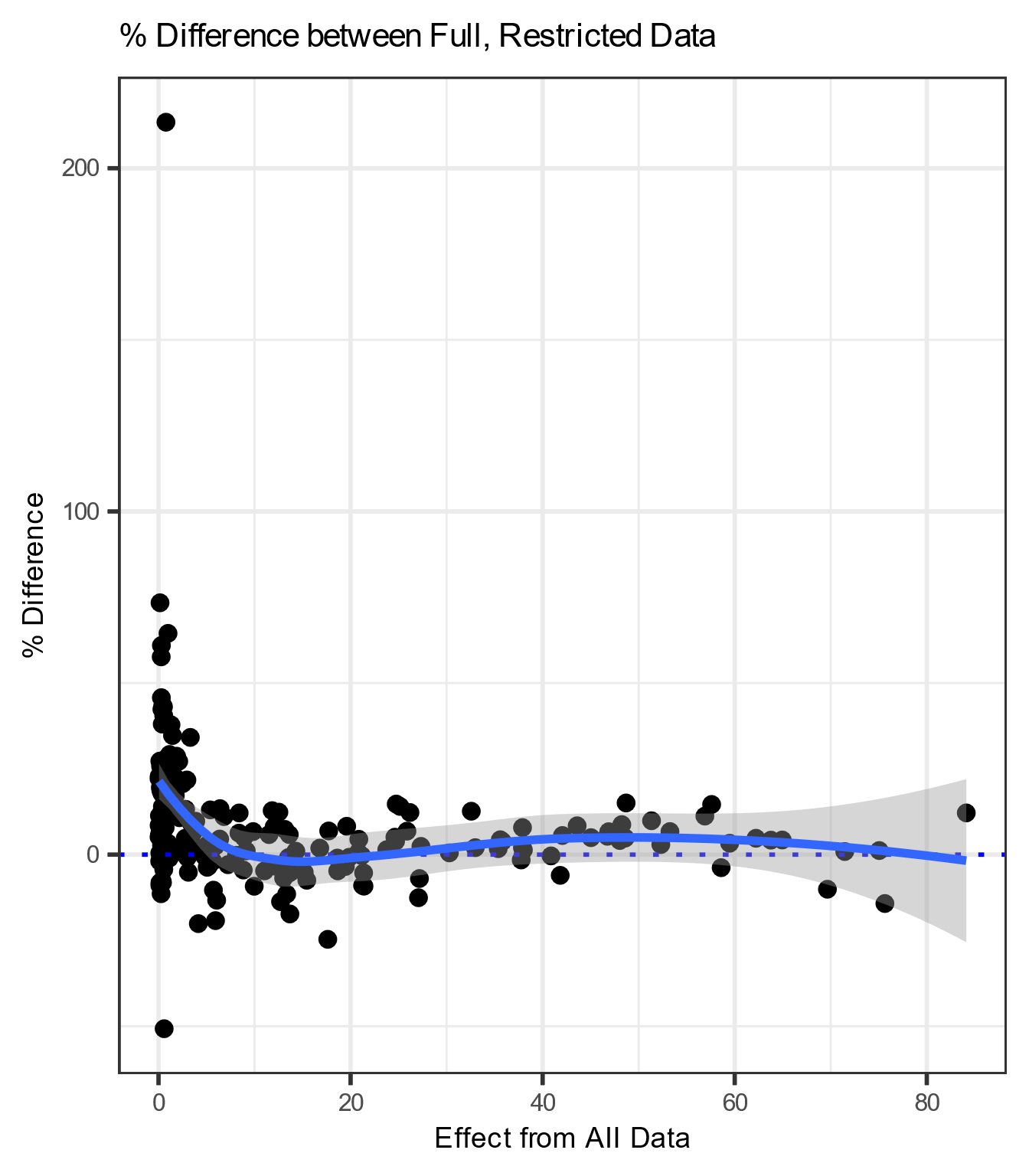
**

Figure S4e. The same data as Figure S4d, but here the difference is plotted as a percentage of the estimate of the effect of from the full dataset.

**Bayesian Model Specification**

We model a chemical’s urine concentration in a sample as an observation, possibly left-censored, from a lognormal distribution whose mean is the sum of contributions from the household, an individual’s product use behaviors, and the cumulative product use behaviors of all other household members. The more usual parameter, the log-median, is then a function of the mean and the estimate of the shape parameter σ.

The contribution of the *k^th^* product use behavior to the mean is the corresponding element of a vector parameter **β** times an indicator variable (a component of a vector ***x***) which has the value 1 if the individual used that product in the previous 24 hours, and 0 otherwise. Product use is split out by gender, so, to use body lotion as an example, the vector ***x*** would have two components, one for females and one for males. Product use behaviors represented by fewer than 10 participants or with fewer than 10 participants NOT represented are dropped. Housemates’ contribution to the overall mean is the product of the sum over all housemates of all products used, normalized so the maximum is 1, times the final component of the vector parameter **β.**

Means for participants from households with more than one representative (households from domain 1) include a household-specific contribution, *Hh_i[j]_*. These are modeled as lognormally distributed values, with unknown (i.e., estimated) log median and shape parameters *σ_A_*_._ Participants from households with single representatives shared a common intercept term. This is summarized as:


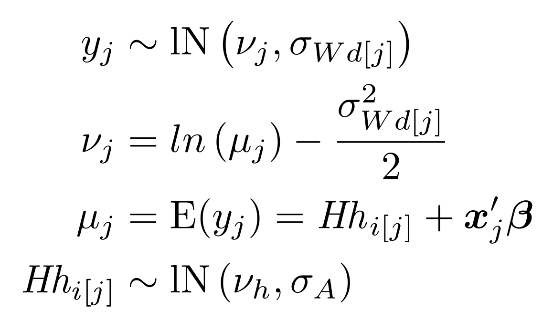


Where *j* indexes participant, *i[j]* is the household for the *j^th^* participant, *d[j]* indicates the domain for participant *j*,  *y_j_* is the urine concentration for the *j^th^* participant, and lN(θ, φ) is the lognormal distribution with log median θ and shape φ.

Parameters were estimated using Bayesian methods and priors:


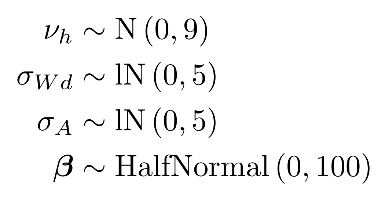


Stan code for full model:

data {

// Data

int N; // number of observations

int K; // number of predictors (may be split into gender-specific columns)

int nAddressGroup; // number of addresses

vector[N] y; // concentrations - either the actual measurement,

// or the LOQ, depending on the value of cens

int cens[N]; // if cens[i] is 0, then y[i] is the actual measurement;

// if cens[i] is 1, y[i] is the LOQ

int newaddressGroup[N]; //integer indicating which household the

//observation belongs to

int domain[N]; // 1 means we have multiple subjects in the house;

// 2 means only a single subject in the house;

// In the latter case, we use a different sigmaW

// predictors

matrix[N, K] x; // product use indicator (0, 1);

// when applicable, the last column is other household members'

// product use

}

parameters {

// Parameters for household-specific background, domain 1

real lsigmaA; //among-household standard deviation

vector[nAddressGroup - 1] lHh0; //normalized log household mean concentration

real nuh; //log mean household background

// Intercept term, domain 2

real lHh2;

// Parameters for individuals

vector[2] lsigmaW; //within-household standard deviation

vector<lower = 0>[K] beta;

}

transformed parameters {

vector[nAddressGroup] Hh; //Contribution to mean of household background

vector[nAddressGroup] lHh; //log of Contribution to mean of household background

vector[N] nuhgx; //lognormal log median for i^{th} individual

real sigmaA;

vector[2] sigmaW;

vector[N] muhgx;

sigmaA = exp(lsigmaA);

sigmaW = exp(lsigmaW);

lHh[1:(nAddressGroup - 1)] = nuh + lHh0 * sigmaA;

lHh[nAddressGroup] = lHh2;

Hh = exp(lHh);

// The log scale parameter for the lognormal is the log of the mean - sigma^2/2

muhgx = Hh[newaddressGroup] + x * beta;

nuhgx = log(muhgx) - (sigmaW[domain] .* sigmaW[domain]) / 2.0;

}

model {

// statements

beta ~ normal(0, 100);

lsigmaA ~ normal(0, 5);

lsigmaW ~ normal(0, 5);

nuh ~ normal(0, 9);

lHh0 ~ normal(0, 1);

// Here we construct the likelihood. This is just censored

// likelihood stuff.

for (i in 1:N) {

target += (cens[i] == 0 ?

lognormal_lpdf(y[i] | nuhgx[i], sigmaW[domain[i]]) :

lognormal_lcdf(y[i] | nuhgx[i], sigmaW[domain[i]]));

}

}

generated quantities {

real hhMean;

real hhMedian;

real yPP[N];

hhMean = exp(nuh + sigmaA * sigmaA / 2.0);

hhMedian = exp(nuh);

yPP = normal_rng(nuhgx, sigmaW[domain]);

}
